# Perceptions of Digital Health Education Among European Medical Students: Mixed Methods Survey

**DOI:** 10.2196/19827

**Published:** 2020-08-14

**Authors:** Felix Machleid, Robert Kaczmarczyk, Doreen Johann, Justinas Balčiūnas, Beatriz Atienza-Carbonell, Finn von Maltzahn, Lina Mosch

**Affiliations:** 1 Association Européenne des Étudiants en Médecine (EMSA) Brussels Belgium; 2 School of Medicine Technical University of Munich Munich Germany; 3 Geography Department Humboldt-Universität zu Berlin Berlin Germany; 4 Lithuanian University of Health Sciences Kaunas Lithuania; 5 Medical Faculty University of Valencia Valencia Spain; 6 Medical Faculty Rheinisch-Westfälische Technische Hochschule Aachen University Aachen Germany; 7 Charité - Universitätsmedizin Berlin (corporate member of Freie Universität Berlin, Humboldt-Universität zu Berlin, and Berlin Institute of Health) Berlin Germany

**Keywords:** medical students, medical education, eHealth, mixed method, health workforce, digital literacy, curriculum

## Abstract

**Background:**

Digital health technologies hold promise to enhance patient-related outcomes, to support health care staff by reducing their workload, and to improve the coordination of care. As key users of digital health technologies, health care workers are crucial to enable a meaningful digital transformation of health care. Digital health literacy and digital skills should become prerequisite competencies for health professionals to facilitate the implementation and leverage the potential of digital technologies to improve health.

**Objective:**

We aimed to assess European medical students’ perceived knowledge and opinions toward digital health, the status of digital health implementation in medical education, and the students’ most pressing needs.

**Methods:**

The explanatory design of our mixed methods study was based on an online, anonymous, self-administered survey targeted toward European medical students. A linear regression analysis was used to identify the influence of the year of medical studies on the responses. Additional analysis was performed by grouping the responses by the self-evaluated frequency of eHealth technology use. Written responses to four qualitative questions in the survey were analyzed using an inductive approach.

**Results:**

The survey received a total of 451 responses from 39 European countries, and there were respondents for every year of medical studies. The majority of respondents saw advantages in the use of digital health. While 40.6% (183/451) felt prepared to work in a digitized health care system, more than half (240/451, 53.2%) evaluated their eHealth skills as poor or very poor. Medical students considered lack of education to be the reason for this, with 84.9% (383/451) agreeing or strongly agreeing that more digital health education should be implemented in the medical curriculum. Students demanded introductory and specific eHealth courses covering data management, ethical aspects, legal frameworks, research and entrepreneurial opportunities, role in public health and health systems, communication skills, and practical training. The emphasis lay on tailoring learning to future job requirements and interprofessional education.

**Conclusions:**

This study shows a lack of digital health-related formats in medical education and a perceived lack of digital health literacy among European medical students. Our findings indicate a gap between the willingness of medical students to take an active role by becoming key players in the digital transformation of health care and the education that they receive through their faculties.

## Introduction

Health care systems around the world are facing challenges connected to an aging population, multimorbidity, an increase of preventable noncommunicable diseases, and health workforce shortages [1-3]. Digital health technologies are seen as a key solution to address these challenges, reinforced by the public health emergency of coronavirus disease 2019 (COVID-19), by having the potential to change the way health services are delivered and promoting the health and well-being of millions of citizens [1,4-6]. For instance, open source technologies have enabled low-cost dissemination and access to data and health information, telehealth technologies have offered communication channels for citizens and health care workers besides physical consultations, and nanotech products have been developed to improve diagnosis and treatment of COVID-19 [7]. However, extensive and sustainable implementation of digital technologies, both into specific clinical settings [8,9] and into national health systems [4,10,11], has been advancing slowly.

Health care professionals play a crucial role in assisting their patients in using digital health technologies appropriately [12,13]. Thus, the need to improve the digital competencies of health workers and citizens to take advantage of digital technologies and facilitate implementation has been emphasized frequently on the international policy level [1,12,14-16].

Major barriers to the successful implementation of digital health technologies are (1) the lack of coordinated, formal education and (2) health care professionals’ skepticism and unwillingness toward implementing digital technologies [17-19]. Engaging with these challenges, medical education, and an effective culture of learning could drive the meaningful digitalization of health care [14]. However, to effectively introduce respective topics in medical education, the needs of key stakeholders and the status of the medical curriculum should be considered.

In this paper, we present and discuss the results of a European-wide study assessing the expectations and needs of medical students regarding digital health competencies and the implementation of digital health in the medical curriculum.

## Methods

### Setting

The European Medical Students’ Association (*Association Européenne des Étudiants en Médecine*; EMSA) is a nonprofit, nongovernmental organization representing the voice of medical students from over 110 faculties in 30 countries across Europe [20]. Data were collected according to the European Union General Data Protection Regulation 2016/679 [21].

### Study Design

We conducted a cross-sectional mixed methods online survey, following an explanatory approach [22-24]. The survey questions were developed during four online discussions after conducting literature research and collecting feedback from external collaborators. We developed 48 questions, of which 29 were quantitative (including 6 questions on demographic data), and 17 were qualitative (see Multimedia Appendix 1).

### Data Collection and Analysis

Data were collected via an informed consent–based online survey in English from June 2018 until August 2018. The target group consisted of medical students in Europe from the first to the seventh year of studies. The survey was distributed via medical faculty mailing lists mapped by EMSA, social media channels, and personal connections.

For the statistical analysis, R (version 3.5.0) and R Studio (version 1.1a) were used [25]. A linear regression analysis was used to identify the influence of the year of medical studies on the responses. Additional analysis was performed by grouping the students depending on the answers to the question “How often are you using eHealth technologies (for example health apps) in your daily life?” *P* values<.05 were deemed statistically significant. For questions where the possible answers ranged from 0 (“strongly disagree”) to 6 (“strongly agree”), the answer options “undecided” and “I do not feel informed enough” were treated adjacently in the linear regression model, as they were situated exactly in the middle of the extremes 0 and 6.

For the linear regression model, the answers “undecided” and “I feel not informed enough” were arbitrarily located adjacent to one another and between the first two and subsequent two options in questions where both these options were included. The dependent variables represented the answers to each question, the independent variable, if not stated otherwise, was the year of medical studies, ranging from first to seventh. Most quantitative questions included an ordinal response format (eg, a Likert-type scale), and in one case, a categorical response format was used. Likert-type scale results were rounded to the next significant digit.

Written responses to four qualitative questions in the survey were analyzed using an inductive approach [26]. The coding and categorization of responses was performed using MaxQDA (version 2020; VERBI GmbH) qualitative data analysis software (Multimedia Appendix 2, Multimedia Appendix 3, Multimedia Appendix 4, and Multimedia Appendix 5) [27]. The results were summarized in paragraphs of continuous text, according to the established code system.

To maintain reflexivity, the research team discussed and established codes and coding in three face-to-face discussions and documented the process of analysis in research diaries throughout the study.

## Results

### Summary

Our study sample consisted of students (n=451) from 39 European countries in all years of medical studies, most aged 18-24 years (344/451, 76.3%). Respondents indicated a need for more eHealth implementation into medical curriculum (agree or strongly agree: 383/451, 84.9%) and a subjective lack of digital skills among the surveyed medical students (evaluated eHealth skills as poor or very poor: 240/451, 53.2%). One quarter (110/451, 24.4%) stated that their faculty provides no eHealth-related courses at all. Surveyed students reported courses on ethical discussions (247/451, 54.8%), research opportunities (211/451, 46.8%), computer science (138/451, 30.6%), usage of eHealth technologies (83/451, 18.4%), start-up possibilities (69/451, 15.3%), and other (36/451, 8.0%).

Qualitatively, we found variability in the responses to the question “Please define eHealth in your own words,” both in the level of detail, scope, and specification. According to the respondents, implementing eHealth in their curriculum would prepare students for their future working environment, keep education up-to-date, and reduce their doubts about eHealth. The already high density of learning content in the medical curriculum was seen as a counterpoint to this. The feeling of being ready to work in a digitized health care system was based on the students’ own technical interests and motivations rather than on adequate training in their faculties. The respondents emphasized being willing to learn about eHealth. They indicated a need for an introduction to eHealth and for specific courses (data management, practical training with eHealth technologies, courses on informatics, ethical aspects, legal frameworks, research and entrepreneurial opportunities, role in public health and health systems, communication skills). Content-wise, the interest lay in learning about recent developments and technologies, health information systems, and artificial intelligence in health. Also, suggestions for cross-disciplinary courses, for teaching eHealth as a separate discipline were made, and that learning should be tailored to future job requirements and interprofessional education.

### Demographic Data

In total, 459 replies were received, of which 2 (0.4%) were left empty and 6 (1.3%) were not willing to participate after reading the initial survey description, resulting in 451 (98.3%) respondents. Our sample was evenly distributed between the first and the sixth year of medical studies. There were fewer seventh-year medical students (22/451, 4.9%) since medical programs with a duration of seven years exist in only a few European countries. The majority of respondents were between 18 and 24 years old (344/451, 76.3%), followed by 25-34 years (98/451, 21.7%). In total, we received responses from 39 countries in the European region with most responses coming from Germany (134/451, 29.0%), Portugal (49/451, 10.9%), and Turkey (39/451, 8.6%).

### Quantitative Results

**Figure 1:**
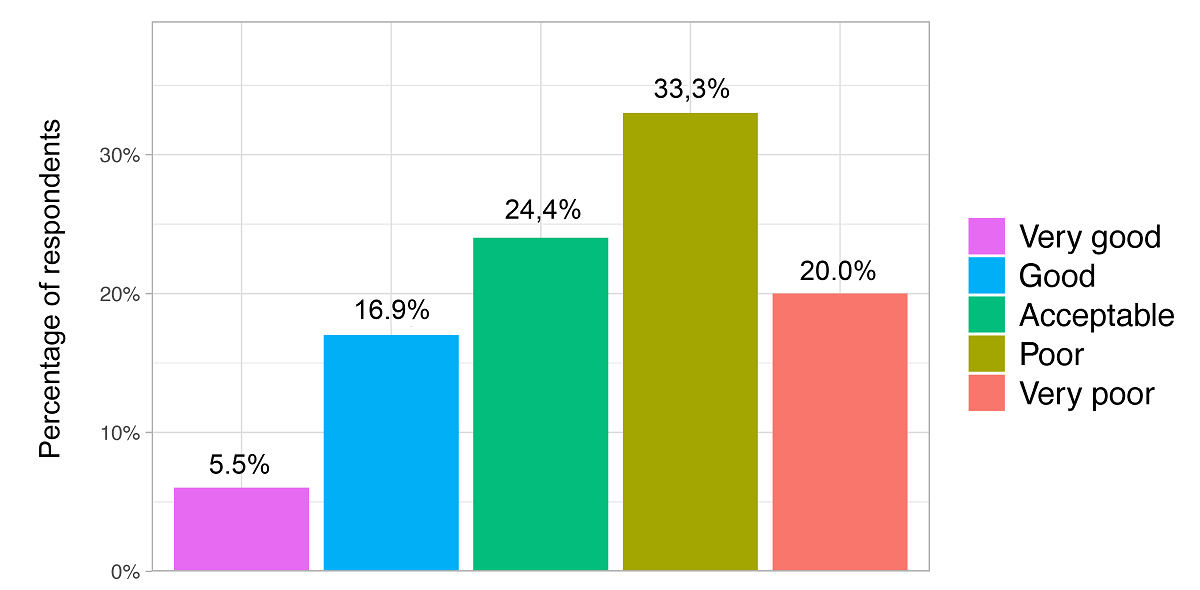
Self-evaluation in response to the statement: "I evaluate my eHealth skills (eg, working with clinical decision support systems, remote patient monitoring systems, artificial intelligence, applications in radiology) as...".

In general, more than half of the respondents (239/451, 53.0%) strongly agreed or agreed on being familiar with the term *eHealth*. Together, almost two-thirds (274/451, 60.8%) of the respondents claimed to never use eHealth technologies or only every other week. Overall, they had positive expectations toward eHealth: they saw mainly or more advantages in mHealth (362/451, 80.3%), telehealth (314/451, 69.6%), and big data (302/451, 67.0%). The respondents strongly agreed or agreed (272/451, 60.3%) that health care professionals should be responsible for eHealth knowledge and skills of their patients. More than half of the respondents (240/451, 53.2%) evaluated their eHealth skills regarding working with eHealth technologies as poor or very poor (Figure 1).

**Figure 2:**
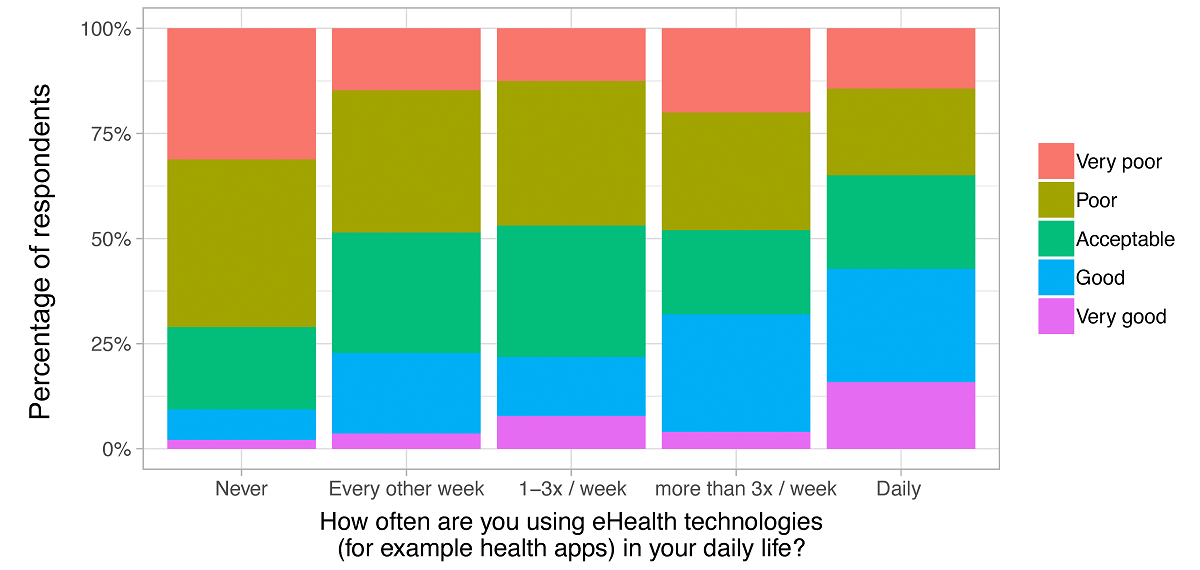
Self-evaluation of the respondents' eHealth skills in relation to their time spent using eHealth technologies in response to the statement: "I evaluate my eHealth skills (eg, working with clinical decision support systems, remote patient monitoring systems, artificial intelligence, applications in radiology) as...".

**Figure 3:**
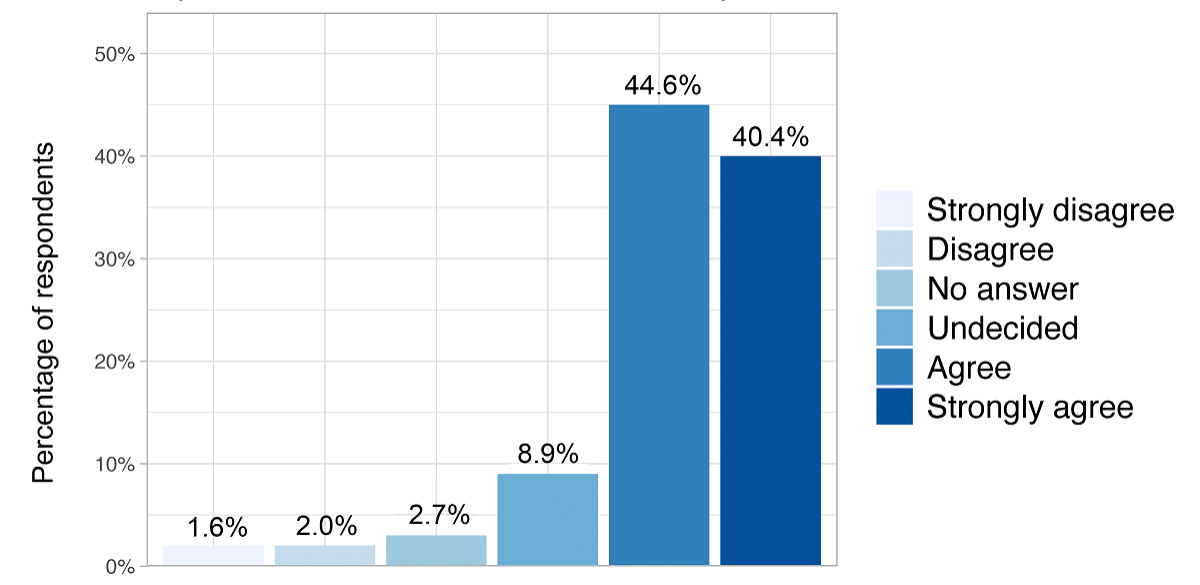
Self-evaluation in response to "Regarding the statement 'I would like eHealth to be more implemented in the medical curriculum,' do you...?".

**Figure 4:**
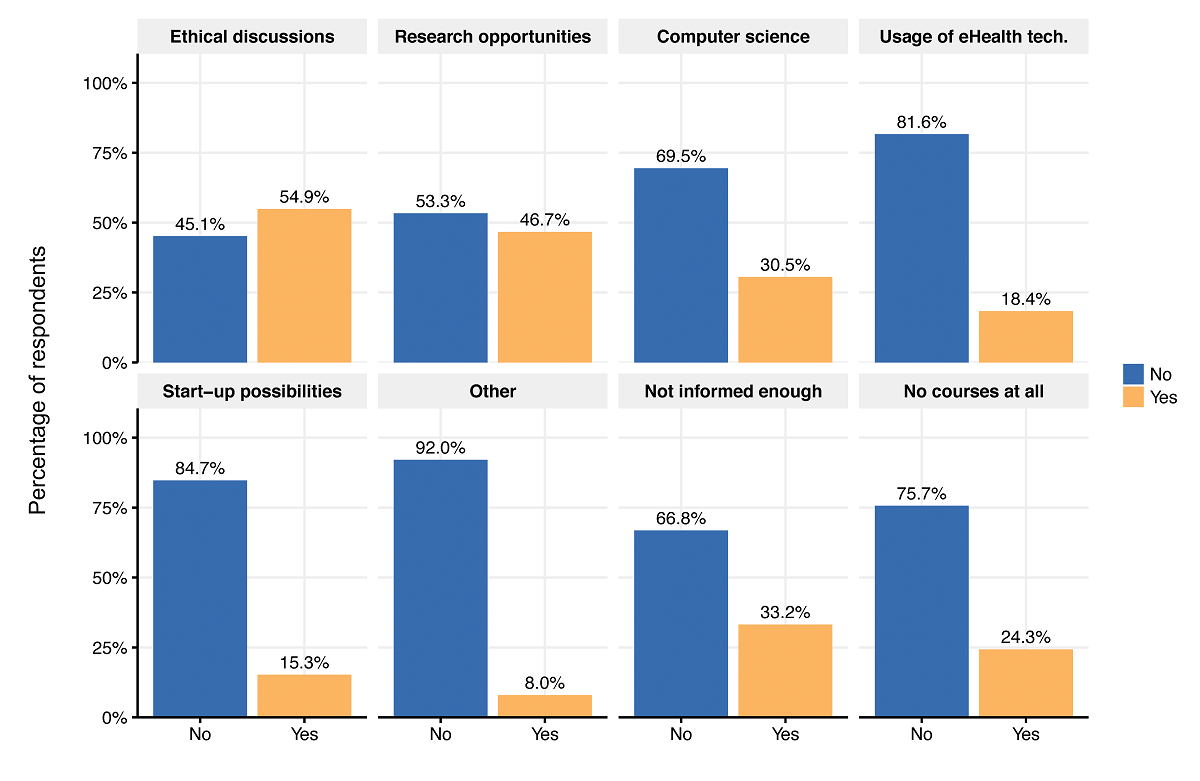
Overview of eHealth-related topics provided by the respondents’ faculty (ie, response to the question: "What eHealth-related topics does your faculty provide courses on?).

Respondents who used eHealth technologies more frequently evaluated their eHealth skills on average as better (*P*<.001) (Figure 2). A majority of respondents (183/451, 40.6%) stated they felt prepared for working in a digitized health care system. Regarding the implementation of eHealth in medical education, we found that 81.8% (369/451) of the students received between 0 and 5 hours of eHealth training during their medical studies. The majority of students (383/451, 84.9%) agreed or strongly agreed that eHealth should be increasingly included in the medical curriculum; 8.9% (40/451) were undecided (Figure 3). Regarding eHealth-related topics provided by the faculty, students stated they received courses on ethical discussions (247/451, 54.8%), followed by research opportunities (211/451, 46.8%), computer science (138/451, 30.6%), usage of eHealth technologies (83/451, 18.4%), start-up possibilities (69/451, 15.3%), and other (36/451, 8.0%). One-quarter (110/451, 24.4%) of the respondents stated that their faculty provided no eHealth-related courses at all. One-third (149/451, 33.0%) said that they were not informed enough to answer this question (Figure 4).

### Qualitative Results

#### Defining eHealth

**Figure 5:**
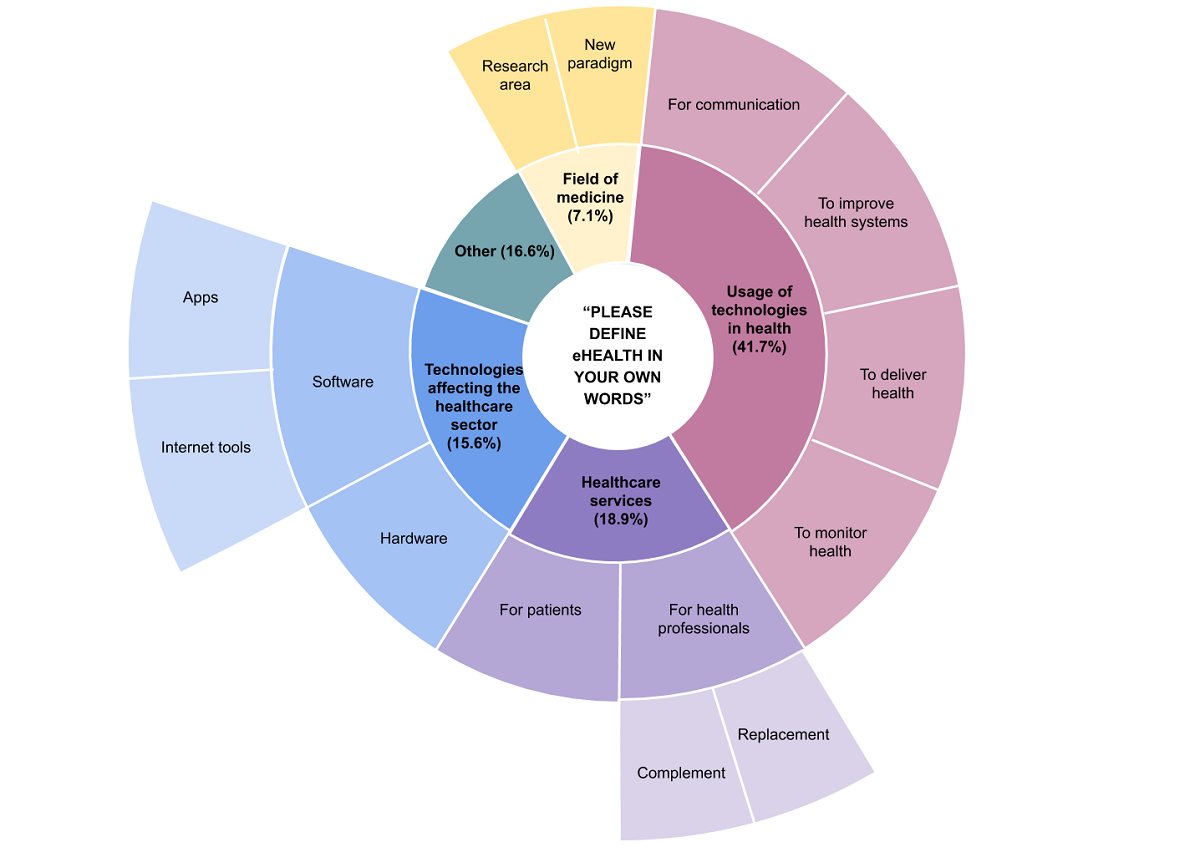
Responses to the statement: "Please define eHealth in your own words." The percentages are in relation to the number of definitions given.

Regarding the task “Please define eHealth in your own words,” 30.3% (137/451) did not give a definition and 1.5% (7/451) admitted not knowing how to define the term *eHealth*. The resulting 68.1% (307/451) provided definitions that were coded and categorized using a grounded theory approach. Of these, 41.7% (128/307) provided a definition coded as *Usage of technologies in health* (Figure 5, Multimedia Appendix 2). The specifications were related to the purpose of technology application in health: (1) delivery of health, (2) monitoring and documentation of health, (3) communication (between health care professionals as well as between doctors and patients), and (4) public health (eg, health promotion, patient-centered health care); 18.9% (58/307) of the definitions fell into the category *Health care services* for patients and health care professionals where eHealth was seen either as a complement to doctors or as a replacement for them (eg, image recognition, clinical decision support systems); 15.6% (48/307) provided definitions of eHealth assigned the code *Technologies affecting the health care sector*, naming examples of software, hardware, internet tools, and health apps. The last category, eHealth as a *Field of medicine* putting digital technologies into practice and research accounted for 7.1% (22/307). The remaining 16.6% (51/307) could not be allocated to any of the preceding categories and fell under the category Other.

#### Reasoning For and Against eHealth in Medical Education

The task “Regarding the statement ‘I would like eHealth to be more implemented in the medical curriculum,’ do you strongly agree, agree, undecided, disagree, strongly disagree? Why?” resulted in a total amount of 151 qualitative answers (151/451, 33.5%). The majority of all respondents (383/451, 84.9%) wished for a stronger implementation of eHealth into the medical curriculum. The reasons provided were that it would prepare them for their future work environment and that learning about eHealth in medical school would be part of keeping the curriculum up-to-date with the latest developments in medicine.

Medicine is a science that should always follow and walk hand by hand with the progress in other resources. Medical school should adapt those changes and provide the best knowledge to the upcoming physicians.

survey response

Furthermore, medical students justified their wish for personal benefits such as digital health literacy as a job qualification and to maintain physicians’ responsibilities and power within the hospital environment and society.

...doctors have to be in charge not IT companies, doctors have to fight for their interest; this is only possible if we have the knowledge and skills...

survey response

Respondents stated that the implementation of eHealth into medical education could decrease the students’ doubts about eHealth technologies. Education on eHealth would drive its effective implementation into health care systems and innovation processes in health. Respondents who were undecided said this was because of a lack of capacities due to an already high workload in medical studies. Some felt that both the quality of education and the resources at their university were not sufficient to sustain the implementation of digital health into the curriculum. Among the reasons for respondents disagreeing were arguments that eHealth topics were already implemented in their curriculum or that digital health literacy should have already been taught in earlier education.

No, this starts much earlier. Dealing with modern technology needs to be taught properly in schools. At university level, students should have basic abilities to deal with ehealth on their own!

survey response

#### Reasons for Feeling or Not Feeling Prepared to Work With eHealth Technologies

The results to the question of whether the respondents felt prepared to work in a digitized health care system (and why or why not) showed mixed opinions. The majority of all respondents (183/451, 40.6%) stated they felt prepared; however, this was not based on adequate training in their faculty but rather on their own technical skills and interests. The respondents agreed that eHealth should be addressed in more medical curricula, both technically-theoretically and practically.

The generation now studying medicine was raised during digitalization so we are very skilled in using its products.

survey response

My ability to use electronic devices and software in general is good, still it would be nice to be prepared for specific software and situations. In university digitization is not considered at all, neither in teaching nor in patient care.

survey response

Respondents not feeling prepared for working in a digitized health care system (130/451, 28.8%) justified this with no or only little eHealth education in their curriculum. They emphasized their willingness to learn more about eHealth.

I haven’t received enough eHealth knowledge or practical skills in order to be able to work appropriately in a digitized health care system. In my opinion, I do not feel completely prepared for working in a digitalized health care system as there is a lack of training in our medical curricula and poor sources in our health care system.

survey response

Respondents who were undecided (129/451, 28.6%) stated that they did not experience any or enough training to evaluate their expertise. They, however, were willing to gain new or extend existing skills:

I’m not sure what a digitized health care system is and I don’t remember if I ever had to interact with it. However, I think it’s something I might be able to handle with my present knowledge.

survey response

#### Opinions on How to Implement eHealth in Medical Education

**Figure 6:**
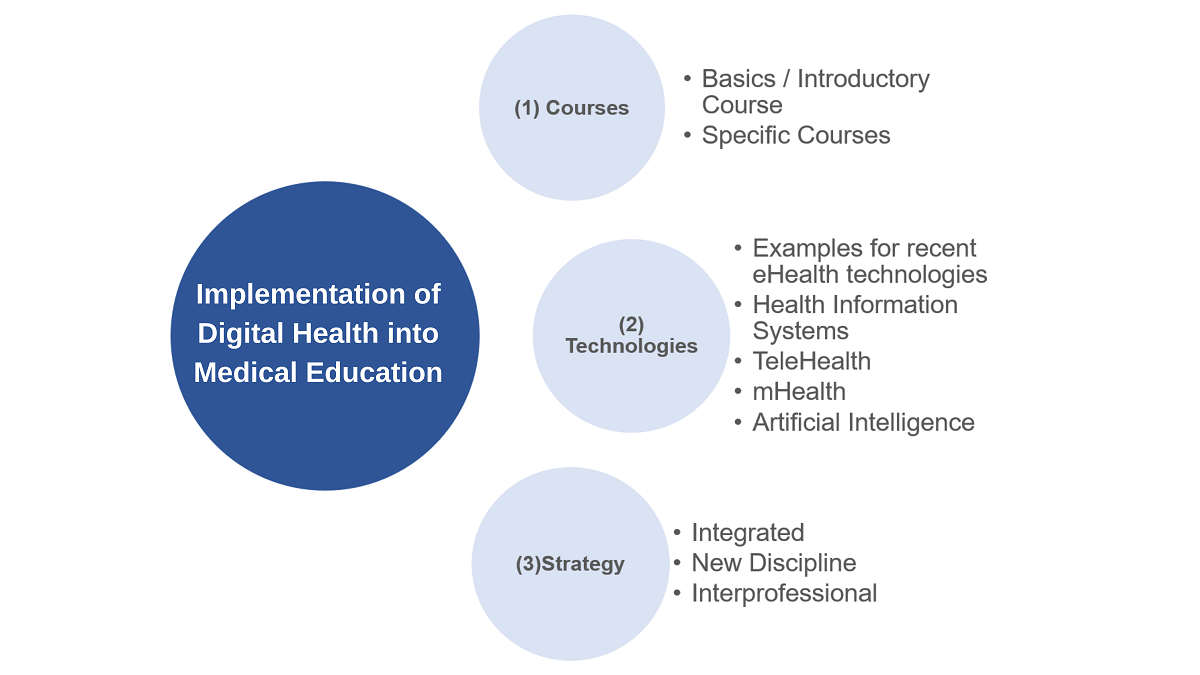
Free text categories derived from responses to the question: "What specific eHealth courses would you like to be implemented in your university’s curriculum?".

The question “What eHealth-related courses would you like to have in your university’s curriculum? (free text)” received 451 answers. Of those, 14.2% (64/451) stated they did not know which courses should be implemented, 9.1% (41/451) gave no answer, and 4.0% (18/451) did not want to have any courses on eHealth. The remaining 72.7% (328/451) were coded and divided into the following categories: Courses, Technologies, and Strategy (Figure 6); hereby, one response could be affiliated with several categories.

Regarding *Courses*, respondents pointed out their need for an introduction to eHealth. For specific courses, education on data management including big data analysis, data sharing, and data security was mentioned most often. Furthermore, practical training with eHealth technologies and courses in computer science (eg, programming languages, app development), the operating principles of eHealth technologies ethical aspects, legal frameworks, research and entrepreneurial opportunities related to eHealth, and the role of eHealth in public health and in health systems were requested. Several respondents wished for courses focusing on communication skills, in particular on how to advise and guide patients using digital health tools. Regarding *Technologies*, respondents wanted to learn about recent examples of eHealth technologies (eg, telehealth and mHealth applications, virtual reality simulations, and robotic applications), health information systems (eg, electronic patient records), and artificial intelligence in health care (eg, clinical decision support systems). Regarding *Strategy* (of implementing eHealth into the medical curriculum), respondents suggested the introduction of cross-disciplinary courses (eg, eHealth in radiology, cardiology), or on the other hand, that eHealth be made a new individual discipline. In both cases, it was suggested that training should be tailored to future job requirements and taught interprofessionally, by involving information technology specialists.

## Discussion

### Principal Findings

The results of this survey illustrated a gap between digital health literacy among medical students and the lack of training and education, despite the willingness of medical students to become key players in the digital transformation of health care. Qualitative analysis of eHealth definitions showed that the student understanding of the subject of eHealth varied considerably. Still, we found all domains of eHealth as described previously by Shaw et al [28] to be covered by the responses. The study showed that student needs in terms of digital health education and training are global and specific; students in the study wished for the implementation of courses ranging from programming languages, legal aspects, and guiding patients using digital health technologies. Students also demanded training with already established health information technology such as health information systems. We found mixed opinions on whether eHealth education should be implemented as a separate discipline or in an integrated form. The respondents highlighted the importance of interprofessional education.

### Digital Health Literacy and Skills for the Future Health Workforce

Among the major factors enabling the successful implementation of eHealth are digital health literacy, digital skills of health care professionals, along with health care professionals’ trust in the potential of novel digital health solutions [9,29]. Furthermore, education and training of health care professionals have been identified as key facilitators of digital health implementation [4,8]. The promotion of these factors requires a multifaceted and diverse approach in order to engage with different stakeholders [1,12,16].

Identifying health care professionals and health care students as central connectors within a challenging digitalization of the health care sector is paramount to its efficacy. In accordance with previous research on the digital skills of health care professionals [12,16,30], our survey showed a gap between the overall willingness of students to become key players in a meaningful digitalization of health care and the competencies and skills they have acquired through their learning.

### Future-Proof Health Care Curriculum

In 2019, the Topol Review [14] identified the top digital technologies affecting 80% of the health workforce in 2040: digital medicine (eg, telemedicine and mHealth), artificial intelligence, robotics, and genomics. Future health care professionals have to become aware of the ethical and patient safety considerations posed by the digital transformation of health care [31]. Additionally, the shift away from technical tasks toward a more patient-centered medicine accompanied by the change in the traditional doctor-patient relationship requires different communication skills [13]. Continuous education and training of health care professionals regarding digital health literacy and skills help to ensure the most effective application of digital health to clinical workflows [32,33]. However, medical students do not necessarily need to become experts in programming and data science as the medical profession will probably continue to be a social one. In line with these considerations and recent publications [14,34,35], the findings of this study indicate that medical education should incorporate basic courses on digital medicine, artificial intelligence in health care, genomics, and data science.

Including the various dimensions of digital health in health curricula is a challenging, yet urgently needed task. Education providers can and should build upon preliminary work [36-41]. Priorities and action plans for the improvement of information technology skills in the EU health care workforce have recently been set out [37].

The recognition of digital skills as key competencies by national training commissions and examination authorities is an essential factor for driving the implementation of this content in health care education [42]. To date, accreditation of digital health literacy and skills in national medical education frameworks is lacking [36,42]. The renowned CanMEDs framework, outlining core competencies for future physicians and serving as orientation for medical educators globally, mentions the term *digital* only in the context of communication and documentation of health data [43]. Our findings support suggestions to revise the CanMEDs and other national frameworks according to the requirements that arise in relation to digital health technologies [13,35,42,44].

Practicing physicians are educators whose proficiency in digital health has a direct impact on the learning outcomes of undergraduates [37]. Therefore, teaching digital health literacy and skills should follow a holistic approach and be integrated into both undergraduate and continuing medical education [45-48].

### Interprofessional Collaboration and International Best Practice Exchange

The creation of future-proof health curricula involves the integration of interprofessional collaboration and supranational coordination and monitoring. Evolving through the disruptive changes brought about by digitalization, health care engages many different professions [44,49]. Topics related to engineering, computer science, and entrepreneurship have become increasingly relevant for medical professionals [49,50]. Our study indicates that medical students are eager to learn interprofessionally and wish to include input from other fields to their education.

In Europe, several networks have been established to support the digitalization of health care and its implementation in health care education [51-54]. Approaches coordinated by the European Commission or EU member states take into account the heterogeneity of European health care systems and the different pace of digitalization in EU member states [16,51]. Additionally, the European exchange of best practices on an institutional level is focused on driving advances in digital health literacy for health care professionals and digital health implementation [14,55]. For instance, platforms such as the European Institute of Innovation and Technology Health, the European Deans’ meeting Training Future-Proof Doctors for the Digital Society, and respective medical education conferences [31,53,56,57] bring together experts and stakeholders in the field. Such platforms for interprofessional collaboration and best practice exchange as well as cross-disciplinary training are essential to ensure continuous improvements in a rapidly changing field.

### Limitations

Our survey was answered by first- to seventh-year medical students throughout Europe. Some of the questions may not have been fully answered by first-year medical students, as some may not yet have had a complete overview of their medical curriculum. Moreover, it is important to mention that the students’ perception of their curriculum might differ from the actual courses offered by their university. The lack of awareness among students suggests faculties need to promote already existing courses.

Our findings may even overestimate the digital health literacy of European medical students. This may be due to two aspects. First, the voluntary mode of participation may suggest that respondents were already aware of and interested in digital health. Thus, it is possible that they do not represent the average knowledge and skill level of medical students regarding digital health and are already more confident about their e-skills abilities than their colleagues would be. Second, people with weak skills tend to overestimate their skills and expected performance [58]. Further research on the eHealth literacy of medical students using validated assessment tools [59,60] would be necessary in order to obtain a clearer and more objective picture.

Due to the limited number of respondents, our results can be considered neither complete nor representative of the European region. Rather, the findings should be seen as a starting point for further research in this area. In a follow-up study, various results could be specifically queried again and linked to current developments in health care education. In addition, findings can be an impetus for policymakers and stakeholders in health care education to revise their approach to meet the needs of future health care professionals with regard to digital skills and eHealth literacy.

The study was conducted in English only, which may have resulted in misunderstandings, inaccuracies, and flawed answers due to a language barrier. Despite the definitions of technical terms given in the survey, their complexity and ambiguity may have caused difficulties in understanding, and subsequently, in answering survey questions. Furthermore, when responding to a Likert scale, it has been shown that respondents were more likely to pick answers in the middle of the scale when responding to an English-language questionnaire compared to a questionnaire in their native language. In turn, questionnaires in a respondents’ native language provoked a higher level of extreme responses [61]. Next to this, the field of eHealth is multidimensional and often not directly labeled as such [28,62], which could have led to misunderstandings among respondents, and thus, biased the survey results.

### Conclusion

This study was the first pan-European approach assessing the needs of medical students regarding digital health literacy and digital skills in medical education. We revealed that the majority of European medical students have a positive attitude toward the digitalization of health care and are willing to play an active role and take responsibility especially as mediators of digital health literacy to patients. However, we also found a lack of knowledge and skills regarding the adequate use and evaluation of digital health technologies, attributed to a lack of respective topics in the medical curriculum. We showed the students’ demand for new, additional teaching concepts ranging from technical-theoretical issues (data management, computer science, legal, and ethical aspects) to practical training with specific technologies and patient communication.

The apparent gap between the overall willingness of medical students to become key players in the digitalization of health care and the education they receive poses a significant challenge to the successful implementation of digital technologies into health care settings. Education providers and policymakers should acknowledge the central role of future health care professionals in health innovation, develop interprofessional concepts ensuring continuous learning, and evaluate them in a continual exchange among themselves and with their students, adapting to the latest scientific and technological developments.

Further research on the specific needs of health care professionals is necessary as new challenges in the growing field of digital health continuously arise. The medical curriculum is essential to create preparatory experiences regarding digital health literacy and digital skills before students enter their professional life. Our findings support the call for faculties and medical education institutions to collaboratively establish targeted, customized, and efficient education and training on digital health.

## Appendix

Multimedia Appendix 1 Catalog of all survey questions.

Multimedia Appendix 2 Codes and codings for "Please define eHealth in your own words.".

Multimedia Appendix 3 Codes and codings for "Regarding the statement 'I would like eHealth to be more implemented in the medical curriculum,' do you... strongly agree, agree, undecided, disagree, strongly disagree, no answer - Why?".

Multimedia Appendix 4 Codes and codings for "Regarding the statement 'I feel prepared for working in a digitized health care system,' do you... strongly agree, agree, undecided, disagree, strongly disagree, no answer - Please elaborate.".

Multimedia Appendix 5 Codes and codings for "What specific eHealth-related courses would you like to have in your university’s curriculum?".

Multimedia Appendix 6 Supplementary figures.

## Abbreviations

EMSA: The European Medical Students’ Association
