# Supplementary material for: Perceptions of Digital Health Education Among European Medical Students: Mixed Methods Survey
Source: J Med Internet Res. 2020 Aug 14;22(8):e19827. doi: 10.2196/19827 (PMC7455864; doi:10.2196/19827)
Supplement: Multimedia Appendix 1 [file jmir_v22i8e19827_app1.pdf]

EMSA survey on eHealth

With the rise of information and communication technology, medicine is being transformed at a pace never seen before. Yet healthcare systems around the globe are struggling to use the full available potential of the newly available tools. Moreover, new knowledge and skills are required in the healthcare workforce, but medical faculties lack vision and are often slow to adopt technology.

The European Medical Students' Association (EMSA) through the European Health Policy Pillar is committed to a advocate for a successful and meaningful digitalization of the healthcare sector and an adequate update of the medical curricula, in frame with the perspective view of the medical students across Europe.

To this end, we have prepared a short survey, where YOU can express your opinion about different aspects of eHealth.

Our aim is to publish the results of the survey; to take a stance with a policy statement and to advocate for our interests as medical students. To realize this we are going to derive recommendations which will be presented to the eHealth stakeholder group of the European Commission and EMSA's external partners (CPME, EHTEL, IFsTeH, EPHA, EPF …).

Kindest regards,
The European Support Division for eHealth

*** IT TAKES AROUND 15 MINUTES TO COMPLETE THE SURVEY.

*Required

Consent

On May 25th 2018, the European Union's General Data Processing Regulation (GDPR) came into effect. The European Medical Students' Association (EMSA), as a registered NGO in a European Union, is naturally subject to this law as well.

The following survey is anonymous so we are not asking for your personal data. To get more information on why and how the data of the survey is collected and processed please refer to this link: https://docs.google.com/document/d/1zi-ExMkBnR8G-laJQcqR4RJ56GrDbu40cJVy1whugYE/edit?usp=sharing

If you have any questions regarding your data collected in this survey please contact policy@emsa-europe.eu
For further questions on EMSAs privacy policies please contact vpc@emsa-europe.eu

1. **Do you agree to take part in this survey sent to you by EMSA?** *

   Mark only one oval.

   - ( ) Yes *Skip to question 2.*
   - ( ) No *Skip to "Thank you!."*

General Questions on eHealth

The questions in the following part are considering general aspects of the eHealth topic.

2. **Regarding the statement "I am familiar with the term of 'eHealth' ", do you... ***

Mark only one oval.

- Strongly agree
- Agree
- Undecided
- Disagree
- Strongly disagree
- No answer

3. **Please define e-Health in your own words**

____

____

____

____

____

4. **How often are you using eHealth technologies (for example health apps) in your daily life? ***

Mark only one oval.

- Daily
- More than 3 times a week
- 1-3 times a week
- Every other week
- Never

The Term of eHealth

eHealth "is the term used to refer to tools and services that use information and communication technologies (ICTs) to improve prevention, diagnosis, treatment, monitoring and management of health and lifestyle."

(https://ec.europa.eu/health/ehealth/overview_en)

NOT TO BE CONFUSED WITH eLearning, which "is learning utilizing electronic technologies to access educational curriculum outside of a traditional classroom. In most cases, it refers to a course, program or degree delivered completely online."

(http://www.elearningnc.gov/about_elearning/what_is_elearning)

5. **Were you aware of the definition of eHealth? ***

Mark only one oval.

- Yes
- No

6. **Were you aware of the differences between eHealth and eLearning? ***

Mark only one oval.

- Yes
- No

Dimensions of eHealth

eHealth encompasses many different aspects. In the following section we will take a closer look at big data, CDSS, telehealth and mHealth.

Wearables and Mobile Apps

Also known as mHealth, are "medical and public health practice supported by mobile devices, such as mobile phones, patient monitoring devices and other wireless devices. Examples: Remote patient monitoring using portable devices like tablets/smartphones; health applications such as Apple Health/Samsung Health/(Apps providing therapeutic options against depression)."

(Global Observatory for eHealth series - Volume 3, World Health Organization)

7. **In the future use of mHealth, I see...** *

Mark only one oval.

- Mainly advantages
- More advantages
- Undecided
- More disadvantages
- Mainly disadvantages
- I feel not informed enough to answer this

8. **Why?**

TeleHealth

TeleHealth is "the use of telecommunications and virtual technology to deliver healthcare outside of traditional health-care facilities. Examples: remote monitoring of vital signs, ECG or blood pressure and remote doctor-patient consultations, through the internet or phone calls."

(definition by the World Health Organisation)

9. **In the future use of teleHealth, I see...** *

Mark only one oval.

- Mainly advantages
- More advantages
- Undecided
- More disadvantages
- Mainly disadvantages
- I feel not informed enough to answer this

10. **Why?**

Big Data

Big Data is "data of a very large size, typically to the extent that its manipulation and management present significant logistical challenges".

(definition by Oxford English Dictionary)

For example, in medicine: administrative claim record, clinical registries, electronic health records, biometric data, patient-reported data, medical imaging, biomarker data, prospective cohort studies, large clinical trials and many others."

11. **In the use of Big Data in future medicine, I see...**
Mark only one oval.

- Mainly advantages
- More advantages
- Undecided
- More disadvantages
- Mainly disadvantages
- I feel not informed enough to answer this

12. **Why?**

Clinical Decision Support Systems (CDSS)

CDSS is a "software that is designed to be a direct aid to clinical decision-making, in which the characteristics of an individual patient are matched to a computerized clinical knowledge base and patient-specific assessments or recommendations are then presented to the clinician or the patient for a decision."

(Sim I, Gorman P, Greenes RA, Haynes RB, Kaplan B, Lehmann H, Tang PC. Clinical Decision Support Systems for the Practice of Evidence-based Medicine. J Am Med Inform Assoc. 2001; Nov-Dec; 8(6): 527–534.)

13. **Regarding the statement "I imagine relying on CDSS in taking decisions in therapy/diagnosis/analyzing and prioritizing data", do you…** *

Mark only one oval.

- Strongly Agree
- Agree
- Undecided
- Disagree
- Strongly Disagree
- I feel not informed enough to answer this

14. **Why?**

Treating Patients

15. **Regarding the statement "Healthcare professionals should be responsible for eHealth knowledge and skills of their patients", do you…** *

Mark only one oval.

- Strongly Agree
- Agree
- Undecided
- Disagree
- Strongly Disagree
- I feel not informed enough to answer this

16. **Why?**

17. **Regarding the statement “Patients should manage their own health data (e.g. laboratory test results, images, health reports)”, do you … ***

Mark only one oval.

- Strongly Agree
- Agree
- Undecided
- Disagree
- Strongly Disagree
- I feel not informed enough to answer this

18. **Why?**

19. **In your opinion, what third parties should have access to patients health data? ***

Mark only one oval per row.

	Yes	No
Health insurance companies		
Pharmaceutical companies		
Doctors / hospital staff		
Universities / other institutions for research purposes		
Other private companies		
Legal / state authorities (police, governments, etc)		
Open Access		
None		
Undecided		
Other		

20. **Please elaborate**

The Future of eHealth

21. **Regarding the statement “eHealth will revolutionize the future of medicine”, do you…** *

Mark only one oval.

- Strongly Agree
- Agree
- Undecided
- Disagree
- Strongly Disagree
- I feel not informed enough to answer this

22. **Why?**

23. **In your opinion: The implementation of eHealth should be regulated and controlled on...** *

Mark only one oval.

- EU-level
- National level (EU member states)
- There is no need for regulation
- Other:

24. **What are your feelings about the future implementation of information and communication technologies into medical practice and research? I feel…** *

Mark only one oval.

	1	2	3	4	5	
Not optimistic						Very optimistic

25. **Why?**

26. **What are your feelings about the future implementation of information and communication technologies into medical practice and research? I feel… (multiple responses possible)** *

Mark only one oval.

	1	2	3	4	5	
Not secure						Very secure

27. **Why?**

eHealth in the Medical Curriculum - Status quo

In the following section we will ask questions regarding the role of eHealth in the medical curriculum and your opinion towards this.

28. **I evaluate my eHealth skills (e.g. working with CDSS, remote patient monitoring systems, Artificial Intelligence (AI), applications in radiology) as...** *

Mark only one oval.

- Very good
- Good
- Acceptable
- Poor
- Very poor

29. **Regarding the statement "My faculty provides lectures/courses on computer literacy and skills (usage of word processing programs, statistical programs, computer based courses)", do you…** *

Mark only one oval.

- Strongly agree
- Agree
- Undecided
- Disagree
- Strongly Disagree

30. **What eHealth-related topics does your faculty provide courses on?** *

Mark only one oval per row.

	Yes	No
Usage of eHealth technologies		
Ethical discussions		
Computer Science (e.g. Data Science, programming languages)		
Research opportunities (e.g. usage of big data)		
Start-up possibilities		
No courses at all		
I feel not informed enough to answer this		
Other		

31. **Please name examples on how eHealth is present in the curriculum at your university. (If it is not present at all write “none”.)** *

32. **Did you/do you participate in eHealth courses at your university?** *
Mark only one oval.

- Yes
- No
- I don't know

33. **How much time of eHealth training did you receive in total?** *
Mark only one oval.

- 0-5 hours
- 5-20 hours
- 20-40 hours
- More than 40 hours

eHealth in the Medical Curriculum - Identifying Trends

34. **Regarding the statement “I would like eHealth to be more implemented in the medical curriculum”, do you..** *
Mark only one oval.

- Strongly agree
- Agree
- Undecided
- Disagree
- Strongly disagree
- No answer

35. **Why?**

36. **What specific eHealth-related courses would you like to have in your university's curriculum?** *

37. **Regarding the statement "eHealth decreases the need of basic clinical and laboratory skills", do you...** *

Mark only one oval.

- Strongly agree
- Agree
- Undecided
- Disagree
- Strongly disagree
- No answer

38. **Why?**

eHealth literacy

eHealth literacy is "the ability to seek, find, understand, and appraise health information from electronic sources and apply the knowledge gained to addressing or solving a health problem".

(Norman CD, Skinner HA. eHealth Literacy: Essential Skills for Consumer Health in a Networked World. Journal of Medical Internet Research. 2006;8(2):e9. doi:10.2196/jmir.8.2.e9.)

39. **According to the definition provided above, my eHealth literacy is...** *

Mark only one oval.

- Very good
- Good
- Acceptable
- Poor
- Very poor

40. **Regarding the statement “I feel prepared for working in a digitized healthcare system.”, do you…** *

Mark only one oval.

- Strongly agree
- Agree
- Undecided
- Disagree
- Strongly disagree
- No answer

41. **Please elaborate**

Last comments

42. **Do you have any other thoughts about eHealth?**

Personal Information

43. **Please select your country of residence:** *

Mark only one oval.

- Albania
- Andorra
- Austria
- Azerbaijan
- Belarus
- Belgium
- Bosnia and Herzegovina
- Bulgaria
- Croatia
- Cyprus
- Czech Republic
- Denmark
- Estonia
- Finland
- France
- Georgia
- Germany
- Greece
- Hungary
- Iceland
- Ireland
- Israel
- Italy
- Kosovo
- Latvia
- Lithuania
- Luxembourg
- Malta
- Monaco
- Montenegro
- Netherlands
- Norway
- Poland
- Portugal
- Moldova
- Romania
- Russian Federation
- San Marino
- Serbia
- Slovakia
- Slovenia

- Spain
- Sweden
- Switzerland
- The former Yugoslav Republic of Macedonia
- Turkey
- Ukraine
- United Kingdom

44. **Please name your city of residence:** *

45. **Please name the university you are currently enrolled at:** *

46. **Please select the year of medical studies:** *
Mark only one oval.

- 1
- 2
- 3
- 4
- 5
- 6
- 7

47. **Please select your age:** *
Mark only one oval.

- 12-17 years old
- 18-24 years old
- 25-34 years old
- 35-44 years old
- 45-54 years old
- 55-64 years old
- 65-74 years old
- 75 years or older

48. **Please select your gender:** *
Mark only one oval.

- Female
- Male
- Prefer not to say
- Other: ______________________

Thank you!

Thank you for taking the time to complete this survey. We truly value the information you have provided. Your responses will contribute to our analyses and suggest new recommendations we as medical students can express.

If you have any comments on the survey or the project please contact policy@emsa-europe.eu

Many thanks,
The European Support Division for eHealth

Powered by
Google Forms
