# Supplementary material for: Perceptions of Digital Health Education Among European Medical Students: Mixed Methods Survey
Source: J Med Internet Res. 2020 Aug 14;22(8):e19827. doi: 10.2196/19827 (PMC7455864; doi:10.2196/19827)
Supplement: Multimedia Appendix 2 [file jmir_v22i8e19827_app2.pdf]

“Please define eHealth in your own words - [free text]"

Color	Code	Segment
●	DEFINITION\Definition eHealth\USAGE of technologies in health\usage of ICT in health\usage of the internet\Internet of	internet of 'medical' things, interconnected internet usage in medicine
●	DEFINITION\Definition eHealth\USAGE of technologies in health\usage of ICT in health\usage of the internet\Internet of	smart technologies in health system
●	DEFINITION\Definition eHealth\USAGE of technologies in health\usage of ICT in health\usage of the internet\Internet of	Using new technology like smart learning systems to improve our health care system
●	DEFINITION\Definition eHealth\USAGE of technologies in health\usage of ICT in	Health care practice using the Internet
●	DEFINITION\Definition eHealth\USAGE of technologies in health\usage of ICT in	Information about health issues on he internet.
●	DEFINITION\Definition eHealth\USAGE of technologies in health\usage of ICT in	Enhancement of medical and health area through technology and internet.
●	DEFINITION\Definition eHealth\USAGE of technologies in health\usage of ICT in	Providing health care using the electronic devices and the Internet.
●	DEFINITION\Definition eHealth\USAGE of technologies in health\usage of ICT in	Providing healthcare or prevent illness by using the internet/technical devices
●	DEFINITION\Definition eHealth\USAGE of technologies in health\usage of ICT in	using internet resources in the practice of medicine
●	DEFINITION\Definition eHealth\USAGE of technologies in health\usage of ICT in	Software that helps to manage health services
●	DEFINITION\Definition eHealth\USAGE of technologies in health\usage of ICT in health\software	Facilities and software provided in order to facilitate access to health matters (vaccination card, doctor appointments, exam results, ...)
●	DEFINITION\Definition eHealth\USAGE of technologies in health\usage of ICT in health	the use of information technologies to access information regarding health
●	DEFINITION\Definition eHealth\USAGE of technologies in health\usage of ICT in health	The use of electronic devices and IT in the framework of health, health services and medicine.
●	DEFINITION\Definition eHealth\USAGE of technologies in health\usage of ICT in health	use of information and communication technologies (apps/internet) to monitor and promote health
●	DEFINITION\Definition eHealth\USAGE of technologies in health\usage of ICT in health	everything that somehow uses technology and informatics or AI in medicine and healthcare
●	DEFINITION\Definition eHealth\USAGE of technologies in health\usage of ICT in health	eHealth is using information and communication technologies for health.

●	DEFINITION\Definition eHealth\USAGE of technologies in health\usage of ICT in health	eHealth is the use of electronic devices or systems and the internet/telecommunications to help give better care to patients in and out of the hospital environment.
●	DEFINITION\Definition eHealth\USAGE of technologies in health\usage of ICT in health	Using modern technology to care about health
●	DEFINITION\Definition eHealth\USAGE of technologies in health\usage of ICT in health	eHealth encompasses all information and communication technologies related to health and wellbeing
●	DEFINITION\Definition eHealth\USAGE of technologies in health\usage of ICT in health	It's the use of information and communication technologies in every aspect of Health industry
●	DEFINITION\Definition eHealth\USAGE of technologies in health\usage of ICT in health	Using information technology to aid health and the health care system
●	DEFINITION\Definition eHealth\USAGE of technologies in health\usage of ICT in health	The application of information technology to any degree in the medical field.
●	DEFINITION\Definition eHealth\USAGE of technologies in health\usage of ICT in health	e-health is an umbrella termin encompassing technological chances brought to medicine and health care delivery by the of modern computing devices and digital infrastructure
●	DEFINITION\Definition eHealth\USAGE of technologies in health\usage of ICT in health	Medical communication and information thanks to the use of new technology and IT devises
●	DEFINITION\Definition eHealth\USAGE of technologies in health\usage of ICT in health	E-Health uses information technology to simplify and improve medical services e.g. telemedicine, electronic health card.
●	DEFINITION\Definition eHealth\USAGE of technologies in health\usage of ICT in health	Electronic and information technologies aiding in prevention, diagnosis and therapy of diseases and organization of medical facilities.
●	DEFINITION\Definition eHealth\USAGE of technologies in health\usage of ICT in health	The way I understand e-Health is the use of information technology in the medical procedures as well as healthcare in general. That could include electronic prescriptions and health records as well as telemedicine.
●	DEFINITION\Definition eHealth\USAGE of technologies in health\usage of ICT in health	Electronic Health: Everything connected to IT and communication devices, which is used in the health sector, e.g.for diagnosis, monitoring, getting information on a health status etc., e.g. Health Apps, telemedicine, electronic health insurance card
●	DEFINITION\Definition eHealth\USAGE of technologies in health\usage of ICT in health	eHealth for me is the usage of digital technology for medicine, medical studies and health care
●	DEFINITION\Definition eHealth\USAGE of technologies in health\usage of ICT in health	ICT applications in health systems - Digital Health (Apps are only a tiny part of it)
●	DEFINITION\Definition eHealth\USAGE of technologies in health\usage of ICT in health	Using telecomunication tools and ICT technology to support/improve the health of the population.

●	DEFINITION\Definition eHealth\USAGE of technologies in health\usage of ICT in health	The use of electronic device in health management.
●	DEFINITION\Definition eHealth\USAGE of technologies in health\usage of ICT in health	The application of electronic devices or applications for the benefit of personal or public health.
●	DEFINITION\Definition eHealth\USAGE of technologies in health\usage of ICT in health	Use of electronic resources to help improve or maintain health
●	DEFINITION\Definition eHealth\USAGE of technologies in health\usage of ICT in health	E-health is the use of electronic devices or resources to manage your health.
●	DEFINITION\Definition eHealth\USAGE of technologies in health\usage of ICT in health	Refers to Health using electronic devices, like apps from mobile phones
●	DEFINITION\Definition eHealth\USAGE of technologies in health\usage of ICT in health	Improving health care by using electronic devices.
●	DEFINITION\Definition eHealth\USAGE of technologies in health\usage of ICT in health	Health through computers and mobile devices
●	DEFINITION\Definition eHealth\USAGE of technologies in health\usage of ICT in health	Healthcare that focuses in the therapy, diagnosis, prevention and documantation on digital possibilities
●	DEFINITION\Definition eHealth\USAGE of technologies in health\usage of ICT in health	EHealth is any kind of digital way to support medical treatment, going from digital wards saving the patient's data to telecommunication with a doctor.
●	DEFINITION\Definition eHealth\USAGE of technologies in health\usage of ICT in health	To use digital solutions (most of them requiring web access) to help improve Medicine, it being by a bigger collection of data or improving the reachability of medical care
●	DEFINITION\Definition eHealth\USAGE of technologies in health\usage of ICT in health	It's the use of electrinc aplications like mobile applications and/or the internet in healthcare and prevention.
●	DEFINITION\Definition eHealth\USAGE of technologies in health\usage of ICT in health	E-Health means to implicate new electronic and digital innovations in the public health system to improve the supply and therapy of diseases.
●	DEFINITION\Definition eHealth\USAGE of technologies in health\usage of ICT in health	Everything what consists of digital medical application.
●	DEFINITION\Definition eHealth\USAGE of technologies in health\usage of ICT in health	use of digital devices in medical and health system
●	DEFINITION\Definition eHealth\USAGE of technologies in health\usage of ICT in health	Everything related to digital tools(equipment, programs, mhealth) that concerns health
●	DEFINITION\Definition eHealth\USAGE of technologies in health\usage of ICT in health	practicing medicine with the help of apps, online platforms and teaching videos

●	DEFINITION\Definition eHealth\USAGE of technologies in health\usage of ICT in health	Use of telemedicine, use of information processing, providing data in clouds, sharing of patient information across different stakeholder groups in medical sector, support in medical services as imaging/diagnoses/administration/insurance/research evidence etc.
●	DEFINITION\Definition eHealth\USAGE of technologies in health\usage of ICT in health	Using digital systems to help patients / myself for a better health.
●	DEFINITION\Definition eHealth\USAGE of technologies in health\usage of ICT in health	everything that digital apps, forms, ways is used in health care, is e-Health
●	DEFINITION\Definition eHealth\USAGE of technologies in health\usage of ICT in health	use of digital technologies in healthcare
●	DEFINITION\Definition eHealth\USAGE of technologies in health\usage of ICT in health	Trying to impact health through social media or web sites and apps.
●	DEFINITION\Definition eHealth\USAGE of technologies in health\usage of ICT in health	the usage of new media and digital tools for patient care, public health etc.
●	DEFINITION\Definition eHealth\USAGE of technologies in health\usage of ICT in health	Using technology to monitor health
●	DEFINITION\Definition eHealth\USAGE of technologies in health\purpose\monitoring	To record or keep track of our health or habits we use certain health apps.
●	DEFINITION\Definition eHealth\USAGE of technologies in health\purpose\monitoring health	e-Health is the use of technology to maintain records of patients as well as to receive information regarding patients in a more convenient manner
●	DEFINITION\Definition eHealth\USAGE of technologies in health\purpose\monitoring	use of technology to assist in tracking health parameters
●	DEFINITION\Definition eHealth\USAGE of technologies in health\purpose\monitoring	Using technology in collecting health related information (?)
●	DEFINITION\Definition eHealth\USAGE of technologies in health\purpose\monitoring	Surveillance of health by personal electronic devices
●	DEFINITION\Definition eHealth\USAGE of technologies in health\purpose\monitoring	Using and storing health related information on eletronic databases
●	DEFINITION\Definition eHealth\USAGE of technologies in health\purpose\delivering	using new technologies to improve health delivery systems
●	DEFINITION\Definition eHealth\USAGE of technologies in health\purpose\delivering	delivering health services via means of virtual media, technology, communication
●	DEFINITION\Definition eHealth\USAGE of technologies in health\purpose\delivering	Providing healthcare with added help by of and through the use of modern information technology.
●	DEFINITION\Definition eHealth\USAGE of technologies in health\purpose\delivering	the use of technologies in the medical professions.

●	DEFINITION\Definition eHealth\USAGE of technologies in health\purpose\delivering	Use of technology in delivering healthcare
●	DEFINITION\Definition eHealth\USAGE of technologies in health\purpose\delivering	The use of internet in order to facilitate the work of doctors and other health professionals.
●	DEFINITION\Definition eHealth\USAGE of technologies in	Communication between patients and doctors via the internet and using health apps
●	DEFINITION\Definition eHealth\USAGE of technologies in health\purpose\communication	Patients data collected and shared throug the database. Almost communications are done by means of electronic devices
●	DEFINITION\Definition eHealth\USAGE of technologies in health\purpose\application in	eHealth is the use of digital technologies in the public health sector
●	DEFINITION\Definition eHealth\USAGE of technologies in health\purpose\application in	using technology to improve the health system of a society
●	DEFINITION\Definition eHealth\USAGE of technologies in health	Providing Health care by using new technology of the industry 3.0 and 4.0
●	DEFINITION\Definition eHealth\USAGE of technologies in health	use of technologies to improve all aspect of health
●	DEFINITION\Definition eHealth\USAGE of technologies in health	e-Health represents the use of technology and electronics with a goal of improving global health
●	DEFINITION\Definition eHealth\USAGE of technologies in health	expanding the usage of technical mediums/resources in the health sector
●	DEFINITION\Definition eHealth\USAGE of technologies in health	Using internet and related technologies like phone applications in healthcare practise.
●	DEFINITION\Definition eHealth\USAGE of technologies in health	Health using computer-like technology
●	DEFINITION\Definition eHealth\USAGE of technologies in health	It's the use of technology on health care. It includes e-descriptions, e-history etc.
●	DEFINITION\Definition eHealth\USAGE of technologies in health	providing health services via the new technologies
●	DEFINITION\Definition eHealth\USAGE of technologies in health	The use of new technologies in medicine
●	DEFINITION\Definition eHealth\USAGE of technologies in health	All the technologies used in medicine I suppose
●	DEFINITION\Definition eHealth\USAGE of technologies in health	technology support in medicine (decision finding, diagnostic, AI Radiology), surveillance of my body parametres
●	DEFINITION\Definition eHealth\USAGE of technologies in health	Using technologies (programs rating X-Ray pictures, communication via internet, portable EEG or ECG programs for smartphones) in field of medecin

●	DEFINITION\Definition eHealth\USAGE of technologies in health	Using technology to improve patient care
●	DEFINITION\Definition eHealth\USAGE of technologies in health	Use of modern technology for health issues, like Apps (patients monitor themselves), teleradiology
●	DEFINITION\Definition eHealth\USAGE of technologies in health	Use of technology in delivering healthcare
●	DEFINITION\Definition eHealth\USAGE of technologies in health	E health is using modern day tec to improve medical services for patients
●	DEFINITION\Definition eHealth\USAGE of technologies in health	The use of technology in health related issues
●	DEFINITION\Definition eHealth\USAGE of technologies in health	The use of digital technology (mainly computers) in the maintenance of health
●	DEFINITION\Definition eHealth\USAGE of technologies in health	use of technology, electronic and comunication to practice medicine and contribute to people's health
●	DEFINITION\Definition eHealth\USAGE of technologies in health	the use of new technologies to improve data analytics and healthcare delivery
●	DEFINITION\Definition eHealth\USAGE of technologies in health	Health care aided by technology.
●	DEFINITION\Definition eHealth\USAGE of technologies in health	The practice of integrating technology into healthcare services and lifestyle medicine. Can include health related websites, applications etc
●	DEFINITION\Definition eHealth\USAGE of technologies in health	use technology to improve the healthcare sytem
●	DEFINITION\Definition eHealth\USAGE of technologies in health	e-Health is the usage of technologies (like applications, electronic devices, etc.) in the field of health (health promotion, health monitoring, treatments,etc.)
●	DEFINITION\Definition eHealth\USAGE of technologies in health	Using technology to improve the medical sector
●	DEFINITION\Definition eHealth\USAGE of technologies in health	Using technology in the interest of health
●	DEFINITION\Definition eHealth\USAGE of technologies in health	using technological tools to improve healthcares
●	DEFINITION\Definition eHealth\USAGE of technologies in health	Healthcare supported by technology
●	DEFINITION\Definition eHealth\USAGE of technologies in health	Health care with the help of new technologies to ease life of patients
●	DEFINITION\Definition eHealth\USAGE of technologies in health	Using technology for different parts of medicine eg diagnosis, communication

●	DEFINITION\Definition eHealth\USAGE of technologies in health	using technology to improve health
●	DEFINITION\Definition eHealth\USAGE of technologies in health	Integration of technology in healthcare.
●	DEFINITION\Definition eHealth\USAGE of technologies in health	Health with the help of technology
●	DEFINITION\Definition eHealth\USAGE of technologies in health	Technology, devices that open new possibilities, connectedness, new information regarding one's health, fitness, well-being
●	DEFINITION\Definition eHealth\USAGE of technologies in health	In my eyes e-Health denotes the use of the internet to research medical knowledge, be it by health professionals or lay persons.
●	DEFINITION\Definition eHealth\USAGE of technologies in health	is the use of internet by health professionals as well as "ignorants" (in the subject of medicine) to clarify any doubt they have about medicine.
●	DEFINITION\Definition eHealth\USAGE of technologies in health	using i.e. technology in our caringsystem. An example would be VR/ these special equipments to see the vessels of the human body without the need of feeling th vessel itself
●	DEFINITION\Definition eHealth\USAGE of technologies in health	e-Health is the collection of digital, mobile and electronic tools and materials with the aim of using them to improve patients safety, public health and application of medicine in different fields.
●	DEFINITION\Definition eHealth\USAGE of technologies in health	Using new technologies in order to improve the diagnosis, management and treatment of chronic diseases
●	DEFINITION\Definition eHealth\USAGE of technologies in health	the use of technology within the medical profession (especially in aspects such as telemedicine)
●	DEFINITION\Definition eHealth\USAGE of technologies in health	Use of electronic equipment to follow up patients
●	DEFINITION\Definition eHealth\USAGE of technologies in health	The use of electronic devices and communication to provide healthcare practices.
●	DEFINITION\Definition eHealth\USAGE of technologies in health	e-Health is aiding of health by electronics
●	DEFINITION\Definition eHealth\USAGE of technologies in health	Healthcare supported by the use of electronic media and data sharing.
●	DEFINITION\Definition eHealth\USAGE of technologies in health	The use of ICT in healthcare.
●	DEFINITION\Definition eHealth\USAGE of technologies in health	usign technology as a tool for medical decisons

●	DEFINITION\Definition eHealth\USAGE of technologies in health	Using internet to reach out more people. Telecommunication of patients and healtcare providers.
●	DEFINITION\Definition eHealth\USAGE of technologies in health	when these electronics play a major role in health data by storing analyzing diagnosis etc
●	DEFINITION\Definition eHealth\USAGE of technologies in health	Use digital resources to collect information and organize therapy for a patient
●	DEFINITION\Definition eHealth\USAGE of technologies in health	The use of technology in patient care, for example online consults and apps
●	DEFINITION\Definition eHealth\USAGE of technologies in health	A simple way to keep track of your disease or to get advice, all With the use of technology
●	DEFINITION\Definition eHealth\USAGE of technologies in health	Zuhilfenahme von Technik in Diagnostik und Überwachung, zum Beispiel um Distanzen zwischen spezialist und Patient zu überbrücken
●	DEFINITION\Definition eHealth\USAGE of technologies in health	Using apps and web pages for improving your health, getting advices etc...
●	DEFINITION\Definition eHealth\USAGE of technologies in health	The use of technology (phone/pc/other electronic devices) to collect and share information about patients in real-time (thanks to internet connection), only if the full respect of the patients privacy can be obtained (e.g.: no sharing data with third parts).
●	DEFINITION\Definition eHealth\USAGE of technologies in health	the use of internet and it's possibilities (videoconference) in health (rehabilitation, at distance apointments, at distance surgery in low income countries)
●	DEFINITION\Definition eHealth\USAGE of technologies in health	use of digital devices and software for study, training and diagnostic
●	DEFINITION\Definition eHealth\USAGE of technologies in health	e-Health is the use of any possible technology in order to stay healthy.
●	DEFINITION\Definition eHealth\USAGE of technologies in health	Use thecnologies than help us to learn or to practice medicine
●	DEFINITION\Definition eHealth\technologies affecting the health sector\E-Health = technologies\Software\internet-tools	internet tools used to prevent, make better or treat various health issues
●	DEFINITION\Definition eHealth\technologies affecting the health sector\E-Health = technologies\Software\internet-tools	Webbased tools supporting health care
●	DEFINITION\Definition eHealth\technologies affecting the health sector\E-Health = technologies\Software\internet-tools	Internet and new tecnology being used to help individuals with health issues

●	DEFINITION\Definition eHealth\technologies affecting the health sector\E-Health = technologies\Software\apps	E-Health is a term for technologies related to health as well as medicine, e.g. applications to control health (body weight, blood pressure etc.) for phones or other devices. It can also refer to tracking and sharing health information in hospitals, private practices etc.
●	DEFINITION\Definition eHealth\technologies affecting the health sector\E-Health =	ehealth is the term described for all (new) technologies, applications in the health sector in times of digitalization
●	DEFINITION\Definition eHealth\technologies affecting the health sector\E-Health = technologies\Software\apps	Digital devices that support health in different aspects, e.g. apps for healthier lifestyle, but also no approaches for physicians to take care for their patients, e.g. via video.
●	DEFINITION\Definition eHealth\technologies affecting the health sector\E-Health =	Computacional programs defined to help health professionals
●	DEFINITION\Definition eHealth\technologies affecting the health sector\E-Health =	Is the set of applications and electronic devices which are used in a medical or health oriented context.
●	DEFINITION\Definition eHealth\technologies affecting the health sector\E-Health =	Any electronic device or program related to health
●	DEFINITION\Definition eHealth\technologies affecting the health sector	Means to affect the health sector by using computers and other electronics
●	DEFINITION\Definition eHealth\technologies affecting the health sector	All sorts of health improving matters that can be found online or through other communicative / technological devices.
●	DEFINITION\Definition eHealth\technologies affecting the health sector	Instruments used to manage medical services or to acces medical information/results as a patient.
●	DEFINITION\Definition eHealth\technologies affecting the health sector	Concepts for treatment, prevention, health (and treatemnt)-tracking and education that are based on electronic devices
●	DEFINITION\Definition eHealth\technologies affecting the health sector	Any digital mean that helps medical professionals to getter all possible information about a pacient or the community
●	DEFINITION\Definition eHealth\technologies affecting the health sector	A system which we can record our health date
●	DEFINITION\Definition eHealth\technologies affecting the health sector	items and systems we as medical staff can use
●	DEFINITION\Definition eHealth\technologies affecting the health sector	Any conseptual electronic related to medicine
●	DEFINITION\Definition eHealth\technologies affecting the health sector	Electronic aids helping in decisionmaking or organisational tasks to improve patient care.
●	DEFINITION\Definition eHealth\technologies affecting the health sector	Apps, Internet, other technologies used to diagnose, stay healthy etc.

●	DEFINITION\Definition eHealth\technologies affecting the health sector	For me eHealth means all the systems that help different hospitals to share their information on patients and the possobility for patients to make appointments online as well as see the health information about themselves as well.
●	DEFINITION\Definition eHealth\technologies affecting the health sector	Tools and applications that track some kind of health metrics (steps, pulse [...])
●	DEFINITION\Definition eHealth\technologies affecting the health sector	It consists of two main subjects. Firstly, apps regarding for example how many steps you've taken or other aspects of daily life. Secondly, sites run by the government or health related institues where one can see one's health data, including doctor's appoints, the tests taken there, as well as the results.
●	DEFINITION\Definition eHealth\technologies affecting the health sector	Every system, application oder function, which supports people staying healthy and doctors doing their jobs
●	DEFINITION\Definition eHealth\technologies affecting the health sector	eHealth "is the term used to refer to tools and services that use information and communication technologies (ICTs) to improve prevention, diagnosis, treatment, monitoring and management of health and lifestyle."
●	DEFINITION\Definition eHealth\technologies affecting the health sector	Everything that help maintain, improwizacja or restore health and is on electronic devices.
●	DEFINITION\Definition eHealth\technologies affecting the health sector	E-Health is a tool to collect and process data in the context of decision making in medicine.
●	DEFINITION\Definition eHealth\technologies affecting the health sector	Telemetric systems that enable continous patient interaction without them being present (if not necessary); promoting health via electronic devices (health apps, brochures, etc.)
●	DEFINITION\Definition eHealth\technologies affecting the health sector	Programms for Medicine/Health
●	DEFINITION\Definition eHealth\technologies affecting the health sector	Every technology health-related
●	DEFINITION\Definition eHealth\technologies affecting the health sector	e-Health for me is all technology related means that someone can use to be upadated about health care issues and also to monitorise their health.
●	DEFINITION\Definition eHealth\technologies affecting the health sector	All electronic and online ressources of prevention, medical support and care.
●	DEFINITION\Definition eHealth\technologies affecting the health sector	digital/electronic technology in health issues

●	DEFINITION\Definition eHealth\technologies affecting the health sector	Any Health application using electronic and digital means for treatment, diagnosis or patient care.
●	DEFINITION\Definition eHealth\technologies affecting the health sector	All technologies regarding health
●	DEFINITION\Definition eHealth\technologies affecting the health sector	e-Health consists of a variety of technologies which are about health. These technologies might be used in hospitals, healthcare units in general and also they can be used by people in their everyday life.
●	DEFINITION\Definition eHealth\technologies affecting the health sector	Digital and new technologies applied to the Healthcare System
●	DEFINITION\Definition eHealth\technologies affecting the health sector	electronic technologies in health care
●	DEFINITION\Definition eHealth\technologies affecting the health sector	All electronic/computer/internet based applications in health services, e.g. patient databases, paperless submission of patient data, online pharmacies, ...
●	DEFINITION\Definition eHealth\technologies affecting the health sector	It means electronic health, which include all the e technology in the aim of health. Starting from the technical electronic equipment for diagnose, applications, etc to communicate, to educate., to diagnose.
●	DEFINITION\Definition eHealth\technologies affecting the health sector	eHealth is a general term for applications and or gadgets which are used by medical staff and patients to compliment the needs in patient care and by healthy people to monitor and change every day health behaviour
●	DEFINITION\Definition eHealth\technologies affecting the health sector	Digital solutions that are used to facilitate work in the health care system, raise awareness, patient education, tools for health care workers etc.
●	DEFINITION\Definition eHealth\technologies affecting the health sector	Data and support technologies people have access to through electrical devices in order to sustain/reach a good condition.
●	DEFINITION\Definition eHealth\technologies affecting the health sector	An electronic system which provides patients and doctors access to traditional and new forms of medical data and possibility to expand the scope of traditional medical care.
●	DEFINITION\Definition eHealth\technologies affecting the health sector	A mordern technology in which individuals can assess their health status electronically and independently.
●	DEFINITION\Definition eHealth\technologies affecting the health sector	technologies used to manage patient's health data in order to extrapolate towards evidence
●	DEFINITION\Definition eHealth\technologies affecting the health sector	the techonology to monitor your health via an electronic environment.

●	DEFINITION\Definition eHealth\technologies affecting the health sector	Technologies used to help people to care more about their health, set their goals and track their progress. Eg, smartphone apps used to track daily steps/sports activity, caloric intake, notes about blood pressure measurements...
●	DEFINITION\Definition eHealth\technologies affecting the health sector	Electronical system for registration and health history of patients that can be shared between hospitals and etc.
●	DEFINITION\Definition eHealth\SERVICES\online-accessable	medical help via social media prgrams
●	DEFINITION\Definition eHealth\SERVICES\online-accessable	Bewareness of Healt in Web/Social Media
●	DEFINITION\Definition eHealth\SERVICES\online-accessable	patient side health applications using modern technology
●	DEFINITION\Definition eHealth\SERVICES\online-accessable Healthcare service\for Healtchare Professionals\assistance, complementation, compensation for medical workforce	Services and products to assist, complement or even compensate a doctor, nurse or any other member of the health providing class that make use of software solutions (sometime in combination with hardware solutions) in providing health services.
●	DEFINITION\Definition eHealth\SERVICES\online-accessable Healthcare service\for Healtchare Professionals\assistance, complementation,	Computer assisted diagnostics and therapy decisions; intelligent documentation tools
●	DEFINITION\Definition eHealth\SERVICES\online-accessable Healthcare service\for Healtchare Professionals\assistance, complementation, compensation for medical workforce	E-Health describes the integration of electronic health data into clinical decision making and a software-based, interconnected approach to diagnostics and treatment to improve quality of care for patients and overall health in the community
●	DEFINITION\Definition eHealth\SERVICES\online-accessable Healthcare service\for Healtchare Professionals\assistance, complementation,	Computer patient documentation
●	DEFINITION\Definition eHealth\SERVICES\online-accessable Healthcare service\for Healtchare Professionals\assistance, complementation,	Substituting any part of the doctor - patient interaction with online resources
●	DEFINITION\Definition eHealth\SERVICES\online-accessable Healthcare service\for Healtchare Professionals\assistance, complementation,	providing and using healthcare data: advancing notices, avoiding complications, prearrangement of resources

•	DEFINITION\Definition eHealth\SERVICES\online-accessable Healthcare service\for Healthchare Professionals\assistance, complementation,	The opportunity to monitor the health of a patient online, so that every professional treating the patient can access information about him/her in a fast way.
•	DEFINITION\Definition eHealth\SERVICES\online-accessable Healthcare service\for Healthchare Professionals\assistance, complementation,	Diagnosis or council Form afar.
•	DEFINITION\Definition eHealth\SERVICES\online-accessable	e-Health is a form of Health Care Service that you can access entirely online.
•	DEFINITION\Definition eHealth\SERVICES\online-accessable	Health services provided on the internet
•	DEFINITION\Definition eHealth\SERVICES\online-accessable	an online health page where you can find information about medicine
•	DEFINITION\Definition eHealth\SERVICES\online-accessable	Health informations and directions available online
•	DEFINITION\Definition eHealth\SERVICES\online-accessable	Médicale information online
•	DEFINITION\Definition eHealth\SERVICES\online-accessable	services in the internet/cloud that support medicine
•	DEFINITION\Definition eHealth\SERVICES\online-accessable	Topics, programs and apps related to health represented on the internet.
•	DEFINITION\Definition eHealth\SERVICES\online-accessable	keeping updated on things regarding health online
•	DEFINITION\Definition eHealth\SERVICES\online-accessable	Online health, through online medical records and a community of online doctors
•	DEFINITION\Definition eHealth\SERVICES\online-accessable	Online services allowing you to monitor your own health, record your health, find information on your health etc
•	DEFINITION\Definition eHealth\SERVICES\online-accessable	Every content on the internet that connected to the healthcare, mental health etc.
•	DEFINITION\Definition eHealth\SERVICES\online-accessable	Apps, online websites collecting data about a medical problem or special illness
•	DEFINITION\Definition eHealth\SERVICES\online-accessable	Health through the internet
•	DEFINITION\Definition eHealth\SERVICES\online-accessable	using the internet and it's webapps to help doctors and patients in any kind of health issues
•	DEFINITION\Definition eHealth\SERVICES\online-accessable	Online platforms for medical advice/appointments/information

•	DEFINITION\Definition eHealth\SERVICES\online-accessable	Internet tools used in everyday working life of a person who works in health section.
•	DEFINITION\Definition eHealth\SERVICES\online-accessable	Health services which are available online?
•	DEFINITION\Definition eHealth\SERVICES\online-accessable	Internet usage in affairs of patient health and treatment
•	DEFINITION\Definition eHealth\SERVICES\online-accessable	E-health is simply the process or processes which include the use of the internet to deliver healthcare services.
•	DEFINITION\Definition eHealth\SERVICES\online-accessable	Health apps and websites
•	DEFINITION\Definition eHealth\SERVICES\online-accessable	Health online
•	DEFINITION\Definition eHealth\SERVICES\online-accessable	accessible and online administration of your personal health
•	DEFINITION\Definition eHealth\SERVICES\online-accessable	providing health services via cyber-methods
•	DEFINITION\Definition eHealth\SERVICES\online-accessable	Access to medical supplies and treatment due to electronic devices.
•	DEFINITION\Definition eHealth\SERVICES\online-accessable	Health services provided eletronically and virtually (without the doctor being in the same room of the patient)
•	DEFINITION\Definition eHealth\SERVICES\ACCESS to health	health data available electronically, available on chip card
•	DEFINITION\Definition eHealth\SERVICES\ACCESS to health	For me it is when you use some mobile apps or programs on your pc that shows how your health is.
•	DEFINITION\Definition eHealth\SERVICES\ACCESS to health related data\personal health data	Propably something to do with patients recieving information about their health via internet and healthcare professionals sharing their information online
•	DEFINITION\Definition eHealth\SERVICES\ACCESS to health	Your health status' availability on the internet.
•	DEFINITION\Definition eHealth\SERVICES\ACCESS to health	E-health is a via internet easy accessible way of collecting information on your own health
•	DEFINITION\Definition eHealth\SERVICES\ACCESS to health	Useful health informations on internet
•	DEFINITION\Definition eHealth\SERVICES\ACCESS to health related data\medical information	Modern way of getting fast tips and tricks on different fields regarding how a person can take care to the best of his abilities his own health... as a profilaxy measure
•	DEFINITION\Definition eHealth\SERVICES\ACCESS to health	Information about health on the internet

●	DEFINITION\Definition eHealth\SERVICES\ACCESS to health	Informations on health available on the Internet instead of by visiting your doctor
●	DEFINITION\Definition eHealth\SERVICES\ACCESS to health	elektronische Informationsquellen oder Möglichkeiten eines direkten Chats mit medizinischem Personal
●	DEFINITION\Definition eHealth\SERVICES\ACCESS to health	Electronic health, technology-related, phone apps to achieve information easily
●	DEFINITION\Definition eHealth\SERVICES\ACCESS to health related data\medical information	eHealth is a way to bring a more advance healthcare system to the people. It is a way of/accessory to medical education, patient care and other daily work, advocacy etc.
●	DEFINITION\Definition eHealth\SERVICES\ACCESS to health related data\both personal health data and medical information	all aspects of medicine that can be transformed into a digital format, that can give rise to huge collections of data which used to be separated and hopefully will benefit us all when studied together
●	DEFINITION\Definition eHealth\SERVICES\ACCESS to health related data	Access to medical or in general information on health issues through electronic devices, without contacting a person who has a lot of experience in the specific area (for example a doctor)
●	DEFINITION\Definition eHealth\SERVICES\ACCESS to health related data	access to our own health data (lab results, reports, etc) and to health knowledge available on the web concerning any medical condition.
●	DEFINITION\Definition eHealth\SERVICES	Services related with health in the digital system: e-appointment, e-recipe, online consultations, digitalization of patient's data, impersonal data for research, apps to follow one's helth, notifications for patients, intersectoral cooperation, e-case histories, etc.
●	DEFINITION\Definition eHealth\SERVICES	Healthcare practice and comunication supported by technology and electronic devices
●	DEFINITION\Definition eHealth\SERVICES	Technological methods (refering to imagistics, medical apps) used to ease the medical process.
●	DEFINITION\Definition eHealth\SERVICES	e-Health is the healthcare services provided over technological devices.
●	DEFINITION\Definition eHealth\SERVICES	electronically assisted health services
●	DEFINITION\Definition eHealth\SERVICES	E-Health can help the doctors to explain and give advice to patients regarding there plan of treatment after a first exploration. This can take place in the Internet.
●	DEFINITION\Definition eHealth\SERVICES	Real life scanning of physical stats and information...
●	DEFINITION\Definition eHealth\Other\We can reach it easily	We can reach it easily

●	DEFINITION\Definition eHealth\Other\Useful	Useful
●	DEFINITION\Definition	technological health
●	DEFINITION\Definition eHealth\Other\relation between health/medicine and technology \integrating medical processes into	E-health is a general term for integrating medical processes into the technology.
●	DEFINITION\Definition eHealth\Other\relation between health/medicine and technology	Combination of medicine and technology as a natural evolution.
●	DEFINITION\Definition eHealth\Other\relation between health/medicine and technology	I don't know, something in relation with medicine and technology
●	DEFINITION\Definition eHealth\Other\relation between health/medicine and technology	Health can collaborated with technology
●	DEFINITION\Definition eHealth\Other\relation between health/medicine and technology	e-health is the health relation with the technologies
●	DEFINITION\Definition eHealth\Other\relation between health/medicine and technology	Health somehow related to the new technologies?
●	DEFINITION\Definition eHealth\Other\relation between health/medicine and technology	Everything connrcted to health and technology
●	DEFINITION\Definition eHealth\Other\relation between health/medicine and technology	Accessing, receiving help and advice with the means of technology.
●	DEFINITION\Definition eHealth\Other\New	New
●	DEFINITION\Definition eHealth\Other\management of one's own	It is the concept of managing your own health through technology such as phone apps, the internet, etc.
●	DEFINITION\Definition eHealth\Other\management of one's own health	Managing healt with the help of modern technology. Can be used in a hospital/proffesional setting, but also as apps on the smartphones.
●	DEFINITION\Definition eHealth\Other\management of one's own	Using technology or web stuff to know the condition of your health, like apps....
●	DEFINITION\Definition eHealth\Other\management of one's own	to use tecnology for healthy life
●	DEFINITION\Definition eHealth\Other\influences on health through	all influences on health through techonology
●	DEFINITION\Definition eHealth\Other\influences on health through	The role and relationship of technology on health
●	DEFINITION\Definition eHealth\Other\influences on health through	I would describe as e-Health the way technology can contribute to the improvement of health serivices
●	DEFINITION\Definition eHealth\Other\influences on health through	Making it easier to share an get Informations

●	DEFINITION\Definition eHealth\Other\influences on health through	promoting health via electronic devices (health apps, brochures, etc.
●	DEFINITION\Definition eHealth\Other\influences on health through	maintaining a healthy lifestyle through use of technology
●	DEFINITION\Definition	Individual medecine
●	DEFINITION\Definition	Healthcare practice
●	DEFINITION\Definition eHealth\Other\Health app initiated lifestyle	Health app initiated lifestyle
●	DEFINITION\Definition eHealth\Other\Health	Health above all
●	DEFINITION\Definition	for instance: step counter
●	DEFINITION\Definition	distance diagnosis and contact
●	DEFINITION\Definition	Electronic Health
●	DEFINITION\Definition eHealth\Other\Electronic Health	e-Health (usually spelled as eHealth) stands for electronic health
●	DEFINITION\Definition eHealth\Other\E-learning in medicine	I thinks it´s a general word for medical things in the internet, like online learning platforms for students, online university courses for students, but also applications for patients that can send their data (RR; HF,..) to their doctors directly.
●	DEFINITION\Definition	Tele medicine
●	DEFINITION\Definition	Telemedicine and personal medicine
●	DEFINITION\Definition eHealth\Other\“The future of healthcare”	“The future of healthcare”
●	DEFINITION\Definition eHealth\Other\“The future of healthcare”	An uptodate and modern way of healthcare
●	DEFINITION\Definition eHealth\Other\"healthy usage" of ICT	Not e-healthy would be internet or game addiction etc.
●	DEFINITION\Definition eHealth\Other\ information (data)	digital information about health in every aspect (knowledge, patient's data, etc)
●	DEFINITION\Definition eHealth\Other\	Virtual health information and statistics for individual
●	DEFINITION\Definition eHealth\FIELD of healthcare/medicine using new technologies\research area on technology in	An area of Medicine that studies the relation between health and technology
●	DEFINITION\Definition eHealth\FIELD of healthcare/medicine using new technologies\new system of medicine	e-Health is the resent healthcare method that supported by technology

●	DEFINITION\Definition eHealth\FIELD of healthcare/medicine using new technologies\new system of medicine	e-Health describes the recent healthcare updated practice via new technology (such as a healthcare database for prescription or a database for taking patients' history or even new applications on smartphones for measuring heartbeat, blood pressure and the levels of glucose) in order to provide more organized and qualitative healthcare system which respects the right of privacy.
●	DEFINITION\Definition eHealth\FIELD of healthcare/medicine using new technologies\new system of medicine	e-Health is a new development in medicine to manage medical problems and patients illness with the help of New technologies
●	DEFINITION\Definition eHealth\FIELD of healthcare/medicine using new technologies\new system of medicine	a system of healthcare through technology
●	DEFINITION\Definition eHealth\FIELD of healthcare/medicine using new technologies\new system of medicine	eHealth is a relatively recent healthcare practice supported by electronic processes and communication
●	DEFINITION\Definition eHealth\FIELD of healthcare/medicine using new technologies\new system of medicine	e-Health is a healthcare practice supported by electronic technologies.
●	DEFINITION\Definition eHealth\FIELD of healthcare/medicine using new technologies\new system of medicine	Traditional health care practice supported by electronic processes to make it faster, more accessible and effective/cost-effective.
●	DEFINITION\Definition eHealth\FIELD of healthcare/medicine using new technologies\new system of medicine	Health care practice that is supported by electronic processes.
●	DEFINITION\Definition eHealth\FIELD of healthcare/medicine using new technologies\new system of medicine	Electronical health system
●	DEFINITION\Definition eHealth\FIELD of healthcare/medicine using new technologies\new system of medicine	new way of thinking of healrh care, updating it with the new internet/electronical sources that are coming out in these last years
●	DEFINITION\Definition eHealth\FIELD of healthcare/medicine using new technologies\new system of medicine	healthcare practice supported by electronic peocesses
●	DEFINITION\Definition eHealth\FIELD of healthcare/medicine using new technologies\new system of medicine	healthcare system assisted by electronic devices, app, etc

●	DEFINITION\Definition eHealth\FIELD of healthcare/medicine using new technologies\new system of medicine	Healthcare practice supported by electronic communication
●	DEFINITION\Definition eHealth\FIELD of healthcare/medicine using new technologies\new system of medicine	Electronically organised health care systems
●	DEFINITION\Definition eHealth\FIELD of healthcare/medicine using new technologies\new system of medicine	medical and public health practice supported by electronic devices, such as internet, patient monitoring devices and other wireless devices.
●	DEFINITION\Definition eHealth\FIELD of healthcare/medicine using new technologies\new system of medicine	e-Health is the health care system in the 21st century, sparing time and money for the professionals and patients as well, and making our work more effective.
●	DEFINITION\Definition eHealth\FIELD of healthcare/medicine using new technologies	It is one of the health branches, which cosists of nowadays software technologies, which can be reached by anyone who has a phone and internet connection
●	DEFINITION\Definition eHealth\FIELD of healthcare/medicine using new technologies	The branch of healthcare which deals with new innovations in online and electronic healthcare.
●	DEFINITION\Definition eHealth\FIELD of healthcare/medicine using new technologies	E-health is a part of the healthsystem where traditional medicin is combined with new electronic means and devices. E.g. Visits at the doctor via skype or using an algorithm to find the right diagnose or therapy.
●	DEFINITION\Definition eHealth\FIELD of healthcare/medicine using new technologies	Area of medicine where the new technologies are exploited
●	DEFINITION\Definition eHealth\FIELD of healthcare/medicine using new technologies	eHealth is an ecosystem regarding all apps that help you track your health, help diagnose diseases and help with the logistics of any given health system.
●	DEFINITION\Definition eHealth\Digitalisation of healthcare sector	Digitalisation of the medical sector
●	DEFINITION\Definition eHealth\Digitalisation of healthcare sector	digitalisation of health care
●	DEFINITION\Definition eHealth\Digitalisation of healthcare sector	Making some healthcare aspects electronical
●	DEFINITION\Definition eHealth\Digitalisation of healthcare sector	e Health concerns all aspects of digitalized medicine and medical care
●	DEFINITION\Definition eHealth\Digitalisation of healthcare sector	Digitalization of health resources
●	DEFINITION\Definition eHealth\Digitalisation of healthcare sector	The digitalisation of medicine and the use of digital tools to improve medical treatment

●	DEFINITION\Definition eHealth\Digitalisation of healthcare sector	digitalizing medicine through using online databases, internet, computers
●	DEFINITION\Definition eHealth\Digitalisation of healthcare sector	E-health is a way to shift health(care) to the digital platform. Through this way everyone is kind of partly their own doctor.
●	DEFINITION\Definition eHealth\Digitalisation of healthcare sector	eHealth is everything concerning digital ways of healthcare-services. This could be an app, a tool for a doctor, for the patients, or concerning the wider ways of health concerning infrastructure.
●	DEFINITION\Definition eHealth\Digitalisation of healthcare sector	eHealth is the digitalization of healthcare and the use of information and communication technologies for health.
●	DEFINITION\Definition eHealth\Digitalisation of healthcare sector	Digitaization of health, including Documentation, payments, prescrption, remote care, patient self care etc.
●	DEFINITION\Definition eHealth\Digitalisation of healthcare sector	E-Health stands for the providence of medical care through an electronic device. This can be done for medication prescription to telemedicine.
●	DEFINITION\Definition eHealth\Digitalisation of healthcare sector	eHealth is the new infrastructure of the health where electronic processes are integrated.
