# Supplementary material for: Perceptions of Digital Health Education Among European Medical Students: Mixed Methods Survey
Source: J Med Internet Res. 2020 Aug 14;22(8):e19827. doi: 10.2196/19827 (PMC7455864; doi:10.2196/19827)
Supplement: Multimedia Appendix 3 [file jmir_v22i8e19827_app3.pdf]

I would like eHealth to be more implemented in the medical curriculum", do you [strongly disagree] [disagree] [undecided] [agree] [strongly agree] - Why?"

Color	Code	Segment
●	Undecided\quality of teaching not good enough	It is very important, but having endured some sdss-courses, I fear the Generation teaching is very unfit for teaching eHealt matters to the Generation studying.
●	Undecided\not enough capacities	Because the physiology, anatomic and medicine knowledge is also important. I don't think I could take much more courses and information.
●	Undecided\not enough capacities	not sure how many courses are still left
●	Undecided\not enough capacities	our curriculum has much bigger problems that should be fixed first
●	No Answer\don't care	I don't really care. Using modern tec is paramount to us anyway, so why bother with curricular stuff?
●	Disagree or Strongly Disagree\total surveillance of citizens	it is a road to total surveillance of the citizens by governments and big companies, whoever is naive enough to go down that road will wake up in 1984
●	Disagree or Strongly Disagree\should be taught in school	No, this starts much earlier. Dealing with modern technology needs to be taught properly in schools. At university level, students should have basic abilities to deal with ehealth on their own!
●	Disagree or Strongly Disagree\not important enough	Not yet important enough
●	Disagree or Strongly Disagree\not important enough	There are way more important topics that should be handled during the study
●	Disagree or Strongly Disagree\already implemented	New curriculum from 2017 at our school already implemented it no need for more.
●	Disagree or Strongly Disagree\already implemented	I think as of now it is well enough implemented.
●	Agree or Strongly Agree\keep physicians' power in society	doctors have to be in charge not IT companies, doctors have to fight for their interest; this is only possible if we have the knowledge and skills
●	Agree or Strongly Agree\keep physicians' power in society	Physicians have to understand what eHealth is and how it is organized in a particular country in order not to drown in fruitless discussions.
●	Agree or Strongly Agree\keep physicians' power in society	It is a necessity and in order to become a slave to it a doctor, you have to master it early. Also a necessity if one really wants to participate in its improvement and implementation.

●	Agree or Strongly Agree\drive eHealth implementation\drive innovation	The technological progess is about to be implemented in every aspect of the medical profession, physicians should be knowledgeable about the methods, structures and possibilities this approach provides.
●	Agree or Strongly Agree\drive eHealth implementation	E-Health is still new and not widely implemented (eospecially in Germnay). Universities should look to encourage innovation and e-health start-ups on their campuses instead of teaching their students how to use applications that will be obsolete once they practice medicine.
●	Agree or Strongly Agree\drive eHealth implementation	Because there is a obvious lack of e-health implementation and in a modern and technologically advance society, e-health is our future.
●	Agree or Strongly Agree\drive eHealth implementation	It is a necessity and in order to become a slave to it a doctor, you have to master it early. Also a necessity if one really wants to participate in its improvement and implementation.
●	Agree or Strongly Agree\decrease doubts and fear of digital health technologies	a lot of students would lose fear of the development towards eHealth by learning about it
●	Agree or Strongly Agree\decrease doubts and fear of digital health technologies	I am very sceptical of future use, mainly because I feel we are not being prepared for it and UMCs are also not prepared for it. I think that increased knowledge will contribute a lot.
●	Agree or Strongly Agree\decrease doubts and fear of digital health technologies	raise awareness, make doctors more sophisticated
●	Agree or Strongly Agree\decrease doubts and fear of digital health technologies	Because it is and will be important for us as medical professionals (if we want or not)!! So we should learn about how to deal with it in a safe and useful manner - cause we then should be the experts, when patients address us with the topic - instead of being afraid of it, cause we don't know it
●	Agree or Strongly Agree\changes quality of healthcare\improvement	This will aid medical students in being better doctors down the line, since they will be equipped with the necessary practice to make full use of eHealth.
●	Agree or Strongly Agree\changes quality of healthcare\improvement	Dealing with data will become ore important and trained skills with ehealth might make work later easier, because you will do less errors and do not have to learn these skills on your own, in your free-time or by learning-on-the-job --> time for actual patient care, efficient

	Agree or Strongly Agree\changes quality of healthcare\improvement	I see a lot of advantages with that.
	Agree or Strongly Agree\changes quality of healthcare\improvement	More automated less errors
	Agree or Strongly Agree\changes quality of healthcare\improvement	Medicine is moving forward with technology.
	Agree or Strongly Agree\changes quality of healthcare\improvement	Medical students must be aware how these disciplines (not only eHealth, but also telemedicine and medical informatics) can help them in their medical practice.
	Agree or Strongly Agree\changes quality of healthcare	As it is definitely going to be part of our future work life I think it is essential that all future physicians have a common base of knowledge, so that all patients have equal access to the different opportunities
	Agree or Strongly Agree\changes quality of healthcare	Its a broader horizon and has lot of potential
	Agree or Strongly Agree\changes quality of healthcare	I believe it will be a game changer in the medical field
	Agree or Strongly Agree\changes quality of healthcare	Because it's our responsibility as future healthcare profeasionals to be informed about the possibility to use information and communication technology for the best opportunities for our patients.
	Agree or Strongly Agree\changes quality of healthcare	Because this is the future, medicine is the only branch of industry that doesn't use customer feedback to improve. I think this can be only changed if we study more about IT (programming languages, data sciences, AI,...)
	Agree or Strongly Agree\changes quality of healthcare	e-Health area seems like the new developed way of our healthcare system. One they when I'm graduated, I will probably find myself in the middle of that system and I don't want to stand there, alone and without enough knowledge.
	Agree or Strongly Agree\changes quality of healthcare	in will be neccessary in future to cope with the increasing medical possibilities and the lack of physicians in rural areas.
	Agree or Strongly Agree\changes quality of healthcare	this is the future; it will help to solve the problem of lacking medical personnel in remote areas
	Agree or Strongly Agree\be prepared\Personal Benefits	Personal benefits
	Agree or Strongly Agree\be prepared\gain knowledge	Because it is such useful knowledge. Why not get access to more and more knowledge?
	Agree or Strongly Agree\be prepared\gain knowledge	To learn what I don't know and my faculty don't teach me

●	Agree or Strongly Agree\be prepared\gain knowledge	It would be helpful to perfect the use of statistical programmes etc.
●	Agree or Strongly Agree\be prepared\gain knowledge	It will strengthen the functionality of medical professionals
●	Agree or Strongly Agree\be prepared\gain knowledge	To get more skills
●	Agree or Strongly Agree\be prepared\gain knowledge	To improve the understanding of students on the practice of health.
●	Agree or Strongly Agree\be prepared\be up to date	I think it is necessary to catch up to the world and current technology
●	Agree or Strongly Agree\be prepared\be up to date	We should follow the new trends of approach
●	Agree or Strongly Agree\be prepared\be up to date	because we need to be more up-to-date with the world around us
●	Agree or Strongly Agree\be prepared\be up to date	We are falling behind of the steam of the time river, ancient methods are dying horribly.
●	Agree or Strongly Agree\be prepared\be up to date	Keeping us up-to-date it's mandatory in medicine
●	Agree or Strongly Agree\be prepared\be up to date	ecause I think that we should learn about the newest trends and opportunities, because after we graduated, our work will be based on this system.
●	Agree or Strongly Agree\be prepared\be up to date	because it will be the future of medicine, if we want it or not, and it would be better to be ahead (or at least at the same Level) as technology's course other than trying to figure it out/ learn about it after it has already taken of the medical field
●	Agree or Strongly Agree\be prepared\be up to date	It's coming anyways...
●	Agree or Strongly Agree\be prepared\be up to date	My university doesn't provide the knowledge or the chance for medical students to be up to date with technologies in medicine
●	Agree or Strongly Agree\be prepared\be up to date	It is always good to be educated as up to date as possible
●	Agree or Strongly Agree\be prepared\be up to date	Medicine is a science that should always follow and walk hand by hand with the progress in other resources. Medical school should adapt those changes and provide the best knowledge to the upcoming physicians.
●	Agree or Strongly Agree\be prepared\be up to date	reform medical teaching for New developments
●	Agree or Strongly Agree\be prepared\be up to date	I want to be in touch with the new technologies and every innovation in the world of medicine.
●	Agree or Strongly Agree\be prepared\awareness about possibilities and risks	as it probably gets more and more important we should learn to handle it and know about its possibilities and risks

●	Agree or Strongly Agree\be prepared\awareness about possibilities and risks	Informed building of opinion
●	Agree or Strongly Agree\be prepared\awareness about possibilities and risks	medical staff has to be familiarized with new technologies and educated about its responsible usage
●	Agree or Strongly Agree\be prepared\awareness about possibilities and risks	In the bachelor it was basically zero, i had one lecture on it, but that was not even a regular course so most people begin their masters with no knowledge about it whatsoever
●	Agree or Strongly Agree\be prepared\awareness about possibilities and risks	cause it is the future so we should know : - how to use it -the good side and the bad side of it -how we can improve it
●	Agree or Strongly Agree\be prepared\awareness about possibilities and risks	Because it's our responsibility as future healthcare profeasionals to be informed about the possibility to use information and communication technology for the best opportunities for our patients.
●	Agree or Strongly Agree\be prepared\awareness about possibilities and risks	raise awareness, make doctors more sophisticated
●	Agree or Strongly Agree\be prepared\awareness about possibilities and risks	It is necessary to understand new technologies to be able to effectively and safely implement them.
●	Agree or Strongly Agree\be prepared\awareness about possibilities and risks	We need to be informed of the possibilities and risks, and we need to discuss ethical issues.
●	Agree or Strongly Agree\be prepared\awareness about possibilities and risks	It is a necessity and in order to become a slave to it a doctor, you have to master it early. Also a necessity if one really wants to participate in its improvement and implementation.
●	Agree or Strongly Agree\be prepared\awareness about possibilities and risks	MDs have to understand the options, benefits and challenges
●	Agree or Strongly Agree\be prepared\ prepare for future work environment	Because it's important to learn what we will be working with in the future.
●	Agree or Strongly Agree\be prepared\ prepare for future work environment	I think that eHealth will revolutionize the healthcare systems so students should be more educated in this area and how they can implement eHealth in their work environment.
●	Agree or Strongly Agree\be prepared\ prepare for future work environment	I would like to get informed and ready about what I'm gonna face when i become a doctor. This is a fair wish.
●	Agree or Strongly Agree\be prepared\ prepare for future work environment	It is important for the future and would be nice to be ahead instead of not learning about it and then not be able to use it.
●	Agree or Strongly Agree\be prepared\ prepare for future work environment	As it is definitely going to be part of our future work life I think it is essential that all future physicians have a common base of knowledge,

●	Agree or Strongly Agree\be prepared\ prepare for future work environment	cause is our future
●	Agree or Strongly Agree\be prepared\ prepare for future work environment	eHealth will play an important role in our future career and we should not have to rely on self-taught skills.
●	Agree or Strongly Agree\be prepared\ prepare for future work environment	Because I believe we are going to have to use these tools in the future inevitably.
●	Agree or Strongly Agree\be prepared\ prepare for future work environment	As an advanced technology it could prove useful in the future therefore medical professionals could benefit from such knowledge.
●	Agree or Strongly Agree\be prepared\ prepare for future work environment	It is a topic with significance for the future and an overview with examples should be given.
●	Agree or Strongly Agree\be prepared\ prepare for future work environment	It is the future. We all know we will get there
●	Agree or Strongly Agree\be prepared\ prepare for future work environment	This is the future
●	Agree or Strongly Agree\be prepared\ prepare for future work environment	Its the future and it requires training
●	Agree or Strongly Agree\be prepared\ prepare for future work environment	It gives a better idea of what is to come during practice
●	Agree or Strongly Agree\be prepared\ prepare for future work environment	it will be important in the future
●	Agree or Strongly Agree\be prepared\ prepare for future work environment	Its the future of medicine.
●	Agree or Strongly Agree\be prepared\ prepare for future work environment	because it will be a big part of my future work
●	Agree or Strongly Agree\be prepared\ prepare for future work environment	If you want it in the clinic you should teach everybody in Uni.
●	Agree or Strongly Agree\be prepared\ prepare for future work environment	It will be very necessary in future medical daily work
●	Agree or Strongly Agree\be prepared\ prepare for future work environment	It's a thing, it's not going to go away so i got to learn about it even if i don't support it
●	Agree or Strongly Agree\be prepared\ prepare for future work environment	Doctors of the future will be working with them.
●	Agree or Strongly Agree\be prepared\ prepare for future work environment	Because it will be used in the futre
●	Agree or Strongly Agree\be prepared\ prepare for future work environment	It’s upcoming so we should be informed and be able to inform patients in the future
●	Agree or Strongly Agree\be prepared\ prepare for future work environment	It will be important in the future.

●	Agree or Strongly Agree\be prepared\ prepare for future work environment	will be very relevant in the future, if we want or not
●	Agree or Strongly Agree\be prepared\ prepare for future work environment	It's the future
●	Agree or Strongly Agree\be prepared\ prepare for future work environment	It is important and will become/is an essential to the daily life of a medical doctor.
●	Agree or Strongly Agree\be prepared\ prepare for future work environment	This is the future
●	Agree or Strongly Agree\be prepared\ prepare for future work environment	That's the future which is used around the world, it is definitely worth to get know it.
●	Agree or Strongly Agree\be prepared\ prepare for future work environment	healthcare workforce needs to be trained, our generation is the one mainly dealing with the digitalisation of healthcare
●	Agree or Strongly Agree\be prepared\ prepare for future work environment	It's the future
●	Agree or Strongly Agree\be prepared\ prepare for future work environment	It's important
●	Agree or Strongly Agree\be prepared\ prepare for future work environment	We will come across it in clinical practice so should be informed about it
●	Agree or Strongly Agree\be prepared\ prepare for future work environment	cause it is the future so we should know : - how to use it -the good side and the bad side of it -how we can improve it
●	Agree or Strongly Agree\be prepared\ prepare for future work environment	ecause I think that we should learn about the newest trends and opportunities, because after we graduated, our work will be based on this system.
●	Agree or Strongly Agree\be prepared\ prepare for future work environment	Because this is the future, medicine is the only branch of industry that doesn't use customer feedback to improve. I think this can be only changed if we study more about IT (programming languages, data sciences, AI,...)
●	Agree or Strongly Agree\be prepared\ prepare for future work environment	e-Health area seems like the new developed way of our healthcare system. One they when I'm graduated, I will probably find myself in the middle of that system and I don't want to stand there, alone and without enough knowledge.
●	Agree or Strongly Agree\be prepared\ prepare for future work environment	It is definitely part of the future of my profession
●	Agree or Strongly Agree\be prepared\ prepare for future work environment	As I previously stated, this is a developing idea that might have a lot of applications in the future
●	Agree or Strongly Agree\be prepared\ prepare for future work environment	It is the future.

●	Agree or Strongly Agree\be prepared\ prepare for future work environment	It will surelx be implemented in the future so as the future of medicine we should be thoroughly taught about the topic
●	Agree or Strongly Agree\be prepared\ prepare for future work environment	It is very important for us, the future doctors, to be competent in every aspect of medicine. Since medicine is shifting towards IT technologies, this is what we have to be knowledgeable in.
●	Agree or Strongly Agree\be prepared\ prepare for future work environment	I think it is a necessary skill to have and is the way forward.
●	Agree or Strongly Agree\be prepared\ prepare for future work environment	It is the future.
●	Agree or Strongly Agree\be prepared\ prepare for future work environment	It's going to be common that people use these devices/apps, so all of us must be prepared and well informed, not only on an optional level.
●	Agree or Strongly Agree\be prepared\ prepare for future work environment	Anything is better than none, especially since it is taking a bigger part of the medical world.
●	Agree or Strongly Agree\be prepared\ prepare for future work environment	because it will be the future of medicine, if we want it or not, and it would be better to be ahead (or at least at the same Level) as technology's course other than trying to figure it out/ learn about it after it has already taken of the medical field
●	Agree or Strongly Agree\be prepared\ prepare for future work environment	It will become very important in the future
●	Agree or Strongly Agree\be prepared\ prepare for future work environment	This is the future. We should learn it.
●	Agree or Strongly Agree\be prepared\ prepare for future work environment	It's a necessary tool for future health professionals.
●	Agree or Strongly Agree\be prepared\ prepare for future work environment	Later eg. at work we could benefit from it.
●	Agree or Strongly Agree\be prepared\ prepare for future work environment	it will become more important
●	Agree or Strongly Agree\be prepared\ prepare for future work environment	It's part of the future!
●	Agree or Strongly Agree\be prepared\ prepare for future work environment	That is obviously where the future is
●	Agree or Strongly Agree\be prepared\ prepare for future work environment	It's coming anyways...
●	Agree or Strongly Agree\be prepared\ prepare for future work environment	It’s what I will have to deal with a lot as soon as I am working!

Agree or Strongly Agree\be prepared\ prepare for future work environment	eHealth is a path that medicine will definitely take, when not in the next few years, than in the next few decades. We will not get aroun it, that's for sure
Agree or Strongly Agree\be prepared\ prepare for future work environment	if it is used in hospitals, medical professionals shall be absolutly capable to use it correctly
Agree or Strongly Agree\be prepared\ prepare for future work environment	Its the future of medicine and if we dont learn how to use it we will not be good specialists.
Agree or Strongly Agree\be prepared\ prepare for future work environment	If that's the future, i would like to know more about it.
Agree or Strongly Agree\be prepared\ prepare for future work environment	It is the future of medicine and each physician should have basic knowledge in this field.
Agree or Strongly Agree\be prepared\ prepare for future work environment	because its the future
Agree or Strongly Agree\be prepared\ prepare for future work environment	going to be mds should know about it
Agree or Strongly Agree\be prepared\ prepare for future work environment	It is important for future doctors to be familiarized with it.
Agree or Strongly Agree\be prepared\ prepare for future work environment	eHealth can't be integrated in medical care if people aren't adequately informed about it or if they don't know how to make use of it. And the best way for those two things to happen is by implementing eHealth in the medical curriculum.
Agree or Strongly Agree\be prepared\ prepare for future work environment	FUTURE
Agree or Strongly Agree\be prepared\ prepare for future work environment	Because there is a obvious lack of e-health implementation and in a modern and technologically advance society, e-health is our future.
Agree or Strongly Agree\be prepared\ prepare for future work environment	Students will have to deal with eHealth and questions from patients about this topic, so they need to be prepared.
Agree or Strongly Agree\be prepared\ prepare for future work environment	in will be neccessary in future to cope with the increasing medical possibilities and the lack of physicians in rural areas.
Agree or Strongly Agree\be prepared\ prepare for future work environment	Its the future
Agree or Strongly Agree\be prepared\ prepare for future work environment	I think it will be part of the healthcare's near future, so I want to be informed and prepared beyond my own personal endeavor.
Agree or Strongly Agree\be prepared\ prepare for future work environment	eHealth is the future of medicine

●	Agree or Strongly Agree\be prepared\ prepare for future work environment	Because that is where I see the future of our job. I believe that this is the necessary step medical treatment needs to take.
●	Agree or Strongly Agree\be prepared\ prepare for future work environment	Training is neccessary to apply them correctly later in practice.
●	Agree or Strongly Agree\be prepared\ prepare for future work environment	Because it is and will be important for us as medical professionals (if we want or not)!! So we should learn about how to deal with it in a safe and useful manner - cause we then should be the experts, when patients address us with the topic - instead of being afraid of it, cause we don't know it
●	Agree or Strongly Agree\be prepared\ prepare for future work environment	EHealth will play a huge part in our future work life, yet we are never taught how to use it in the best way.
●	Agree or Strongly Agree\be prepared\ prepare for future work environment	More and more people will be using it
●	Agree or Strongly Agree\be prepared\ prepare for future work environment	Because It could be the future
●	Agree or Strongly Agree\be prepared\ prepare for future work environment	Because it is a very significant part of my future as a doctor.
●	Agree or Strongly Agree\be prepared\ prepare for future work environment	important in the future
●	Agree or Strongly Agree\be prepared\ prepare for future work environment	Whether we like it or not, we've found a way to make things easier for us and therefore it will most likely be the future
●	Agree or Strongly Agree\be prepared\ prepare for future work environment	Because the majority of students are only faced with programs and stuff after starting their work
●	Agree or Strongly Agree\be prepared\ prepare for future work environment	becaue it is the future
●	Agree or Strongly Agree\be prepared\ prepare for future work environment	Medical students must be aware how these disciplines (not only eHealth, but also telemedicine and medical informatics) can help them in their medical practice.
●	Agree or Strongly Agree\be prepared\ prepare for future work environment	It's the future
●	Agree or Strongly Agree\be prepared\ prepare for future work environment	believe these new generations of doctors should be familiar and comfortable in approaching eHealth
●	Agree or Strongly Agree\be prepared\ prepare for future work environment	lack of preparedness
●	Agree or Strongly Agree\be prepared\ prepare for future work environment	this is the future; it will help to solve the problem of lacking medical personnel in remote areas

●	Agree or Strongly Agree\be prepared\ prepare for future work environment	I want to be safe when rainy day comes. What young doctors will do when they joing health system? Error and trial is a way, but I think its not the most efficient.
●	Agree or Strongly Agree\be prepared\ prepare for future work environment	It will affect us all in the future, which is why we need to be proficient in eHealth from an early stage on.
●	Agree or Strongly Agree\be prepared\ prepare for future work environment	I think future doctors need to know more about a reality that is near.
●	Agree or Strongly Agree	AgreeWe just need it
●	Agree or Strongly Agree	Could be useful
