# Supplementary material for: Perceptions of Digital Health Education Among European Medical Students: Mixed Methods Survey
Source: J Med Internet Res. 2020 Aug 14;22(8):e19827. doi: 10.2196/19827 (PMC7455864; doi:10.2196/19827)
Supplement: Multimedia Appendix 4 [file jmir_v22i8e19827_app4.pdf]

Regarding the statement "I feel prepared for working in a digitized healthcare system", do you… [strongly disagree] [disagree] [undecided] [agree] [strongly agree] - Why?

Code	Segment
Reasons: Undecided\the physician occupation will disappear over time	I am sort of scared that the physician occupation will disappear over time.
Reasons: Undecided\not informed/experienced enough, but I'm motivated	Not informed enough
Reasons: Undecided\not informed/experienced enough, but I'm motivated	I do not have the skills but am willing to learn
Reasons: Undecided\not informed/experienced enough, but I'm motivated	I feel I could learn, but not that I am prepared for it right now
Reasons: Undecided\not informed/experienced enough, but I'm motivated	I feel like it will take me for a while to adapt
Reasons: Undecided\not informed/experienced enough, but I'm motivated	3rd year student, I don't know what is yet to come.
Reasons: Undecided\not informed/experienced enough, but I'm motivated	I haven't been professionally prepared in that regard
Reasons: Undecided\not informed/experienced enough, but I'm motivated	I still need to build my knowledge on eHealth as i just have little knowledge on some parts of it.
Reasons: Undecided\not informed/experienced enough, but I'm motivated	i still need training and getting used to it, but after some time, I think everyone can get the handle of it
Reasons: Undecided\not informed/experienced enough, but I'm motivated	I have not yet had any formal training in this area, but I am prepared to learn on my own.
Reasons: Undecided\not informed/experienced enough, but I'm motivated	Not enough experience to assess my preparedness.
Reasons: Undecided\not informed/experienced enough, but I'm motivated	I'm not sure what a digitized healthcare system is and I don't remember if I ever had to interact with it. However, I think it's something I might be able to handle with my present knowledge.
Reasons: Undecided\not informed/experienced enough, but I'm motivated	First year student (not yet prepared to work at all)
Reasons: Strongly agee / agree\yes, but I need to learn it more	I need to learn more computer literacy
Reasons: Strongly agee / agree\yes, but I need to learn it more	I am very open to and have an affinity to tech that makes my life an work easier. But education and much more adaptation in hospitals should improve. Fast
Reasons: Strongly agee / agree\yes, but I need to learn it more	I probably have gaps in my knowledge but feel sufficiently able to adapt and learn new eHealth skills as necessary

Reasons: Strongly agee / agree\yes, but I need to learn it more	I am not 100% sure how to use each one of them, but I have the motivation to learn it and place it in my everyday work, as it helps me with patients, with diagnosis and treatment, and saves me more time.
Reasons: Strongly agee / agree\yes, but I need to learn it more	I might need some induction but generally I do understand how it works.
Reasons: Strongly agee / agree\yes, but I need to learn it more	I would have to try to know for sure, but I believe I could
Reasons: Strongly agee / agree\yes, but I need to learn it more	I think I need to learn more on data security and laws reglementing the use of eHealth data.
Reasons: Strongly agee / agree\yes, but I need to learn it more	have not had any courses, but as Born in Estonia, where it is a todays life that you have most medical data available in e-Environment, I have some experience
Reasons: Strongly agee / agree\yes, but I need to learn it more	I would manage to work in digitalised ehalth somehow, its not that hard, but I think universities could teach the full capabilities of ehealth and how to apply to daily work.
Reasons: Strongly agee / agree\yes, but I need to learn it more	when given the appropriate introduction courses to software/hardware
Reasons: Strongly agee / agree\yes, but I need to learn it more	I know computers, internet, apps, programing, but can learn more specific stuff
Reasons: Strongly agee / agree\yes, but I need to learn it more	There is stimulus on this field from my university, however I'm feeling there are a lot more to learn, and I believe it will take me some time to catch up
Reasons: Strongly agee / agree\yes, but I need to learn it more	I feel like I could work in such a system however I would need someone to guide me at first.
Reasons: Strongly agee / agree\yes, but eHealth should be a bigger point in med ed	teaching on this area in my med ed was very poor, most things i picked up myself and probably had a good computer literacy allready, but there were no specific trainings to prepare doctors for those tasks
Reasons: Strongly agee / agree\yes, but eHealth should be a bigger point in med ed	I did not learn a lot about eHealth in my medical studies. I just know some basic things about IT in general, so I don't feel well prepared. Otherwise in Germany eHealth (unfortunately) is also still not that big in the hospitals, so it probably will grow together with me and my skills ^^.
Reasons: Strongly agee / agree\yes, but eHealth should be a bigger point in med ed	I would manage to work in digitalised ehalth somehow, its not that hard, but I think universities could teach the full capabilities of ehealth and how to apply to daily work.
Reasons: Strongly agee / agree\yes, but eHealth should be a bigger point in med ed	when given the appropriate introduction courses to software/hardware

Reasons: Strongly agee / agree\yes, but eHealth should be a bigger point in med ed	My ability to use electronic devices and software in general is good, still it would be nice to be prepared for specific software and situations. In university digitization is not considered at all, neither in teaching nor in patient care.
Reasons: Strongly agee / agree\explicit eHealth skills	I have experiance using hospital administration programmes, and I am very much open to these new technologies and waiting for these to be the part of my job to use these
Reasons: Strongly agee / agree\explicit eHealth skills	I am involved in ISfTeH and have worked with such programs: PACS system and OpenMRS.
Reasons: Strongly agee / agree\explicit eHealth skills	I'm a pharmacist (and a PhD student)- I already work with eHealth technologies and love them
Reasons: Strongly agee / agree\explicit eHealth skills	I am currently enrolled in two separate studies approaching university degress in medicine and computer science. I am about to write a doctoral thesis on the application of digital health in the ICU.<br>I am fluent in several programming languages as
Reasons: Strongly agee / agree\explicit eHealth skills	I am actively involved in state eHealth questions through my NGO
Reasons: Strongly agee / agree\explicit eHealth skills	This is so because I have personal carried out a research on adoption of telemedicine in Southwestern Nigeria.
Reasons: Strongly agee / agree\explicit eHealth skills	I am working for many years in this environment and developing the applications.
Reasons: Strongly agee / agree\because of personal knowledge/skills, I grew up in a digitalize	Meanwhile my college has been short on such trainings I have personal experience utilizing electronic sources for clinical knowledge also I have personal experience with many coding languages.
Reasons: Strongly agee / agree\because of personal knowledge/skills, I grew up in a digitalize	The generation now studying medicine was raised during digitalization so we are very skilled in using its products.
Reasons: Strongly agee / agree\because of personal knowledge/skills, I grew up in a digitalize	if i am, it's because of myself not of the faculty
Reasons: Strongly agee / agree\because of personal knowledge/skills, I grew up in a digitalize	I am very open to and have an affinity to tech that makes my life an work easier. But education and much more adaptation in hospitals should improve. Fast
Reasons: Strongly agee / agree\because of personal knowledge/skills, I grew up in a digitalize	I grew up during digitalization, it's easy so search and find on the internet, to use apps and to use it in health care.
Reasons: Strongly agee / agree\because of personal knowledge/skills, I grew up in a digitalize	I am very good with technology and am very interested in it, so I see no major problems for the coming years.

Reasons: Strongly agee / agree\because of personal knowledge/skills, I grew up in a digitalize	focused
Reasons: Strongly agee / agree\because of personal knowledge/skills, I grew up in a digitalize	I am a very nerdy person and I work like a sponge of I hold technology in my hands.
Reasons: Strongly agee / agree\because of personal knowledge/skills, I grew up in a digitalize	My generation have more or less evolved with technology so I feel confident in applying it in my work.
Reasons: Strongly agee / agree\because of personal knowledge/skills, I grew up in a digitalize	using
Reasons: Strongly agee / agree\because of personal knowledge/skills, I grew up in a digitalize	Concering finding and evaluating electronic data, I think the generation 10-30 years is way better prepared compared to those 30+
Reasons: Strongly agee / agree\because of personal knowledge/skills, I grew up in a digitalize	teaching on this area in my med ed was very poor, most things i picked up myself and probably had a good computer literacy allready, but there were no specific trainings to prepare doctors for those tasks
Reasons: Strongly agee / agree\because of personal knowledge/skills, I grew up in a digitalize	I feel that technological skills are not improved during our education; everyone has to learn on his own so there are people that have no skills at all
Reasons: Strongly agee / agree\because of personal knowledge/skills, I grew up in a digitalize	I’m confident with technologies
Reasons: Strongly agee / agree\because of personal knowledge/skills, I grew up in a digitalize	I don't have any difficulties learning a new system or finding information online.
Reasons: Strongly agee / agree\because of personal knowledge/skills, I grew up in a digitalize	Having grown up using technology, I am comfortable with learning to use different healthcare systems easily.
Reasons: Strongly agee / agree\because of personal knowledge/skills, I grew up in a digitalize	This is so because I have personal carried out a research on adoption of telemedicine in Southwestern Nigeria.
Reasons: Strongly agee / agree\because of personal knowledge/skills, I grew up in a digitalize	We all grew up with this stuff, c'mon
Reasons: Strongly agee / agree\because of personal knowledge/skills, I grew up in a digitalize	A lot of the learning in medicine depends on the capability of finding the reports, journals, articles and books with the information you need. Also we grew up online and using eletronics since ever so I think our generation is more than ready to deal with the digitalized word.
Reasons: Strongly agee / agree\because of personal knowledge/skills, I grew up in a digitalize	My ability to use electronic devices and software in general is good, still it would be nice to be prepared for specific software and situations. In university digitization is not considered at all, neither in teaching nor in patient care.
Reasons: Strongly agee / agree\because of education in university	Good university

Reasons: Disagree / Strongly disagree\there is no or less education in university regarding eHealth	I do not get the education on this, I won't be able to use them or understand them. This will reduce my effectiveness in this new era.
Reasons: Disagree / Strongly disagree\there is no or less education in university regarding eHealth	I feel that technological skills are not improved during our education; everyone has to learn on his own so there are people that have no skills at all
Reasons: Disagree / Strongly disagree\there is no or less education in university regarding eHealth	It's actually the first time I hear something about e-health, how could I fell prepared for working through it?
Reasons: Disagree / Strongly disagree\there is no or less education in university regarding eHealth	First I need to be trained. I might say I feel prepared to be trained for working in a digitized helathcare system.
Reasons: Disagree / Strongly disagree\there is no or less education in university regarding eHealth	I am yet to receive enough education to be fully confident.
Reasons: Disagree / Strongly disagree\there is no or less education in university regarding eHealth	I don't believe I got the enough education about eHealth in my faculty.
Reasons: Disagree / Strongly disagree\there is no or less education in university regarding eHealth	I have not had training as yet on the issue.
Reasons: Disagree / Strongly disagree\there is no or less education in university regarding eHealth	i have not had any training in this regard as of yet. i will be open to learning more about it to then work with such systems providing that they do not breach data protection of the patients
Reasons: Disagree / Strongly disagree\there is no or less education in university regarding eHealth	Since I have never had any courses in this field, I dont think I'm well equipped for this
Reasons: Disagree / Strongly disagree\there is no or less education in university regarding eHealth	have not had any courses, but as Born in Estonia, where it is a todays life that you have most medical data available in e-Environment, I have some experience
Reasons: Disagree / Strongly disagree\there is no or less education in university regarding eHealth	they did not teach any of this stuff
Reasons: Disagree / Strongly disagree\there is no or less education in university regarding eHealth	We haven't yet practiced anything regarding ehealth.
Reasons: Disagree / Strongly disagree\there is no or less education in university regarding eHealth	I haven't received enough eHealth knowledge or practical skills in order to be able to work appropriately in a digitized healthcare system.
Reasons: Disagree / Strongly disagree\there is no or less education in university regarding eHealth	More classes please!
Reasons: Disagree / Strongly disagree\there is no or less education in university regarding eHealth	In my opinion, I do not feel completety prepared for working in a digitalized healthcare system as there is a lack of training in our medical curricula and poor sources in our healthcare system.

Reasons: Disagree / Strongly disagree\there is no or less education in university regarding eHealth	I did not learn a lot about eHealth in my medical studies. I just know some basic things about IT in general, so I don't feel well prepared. Otherwise in Germany eHealth (unfortunately) is also still not that big in the hospitals, so it probably will grow together with me and my skills ^^.
Reasons: Disagree / Strongly disagree\there is no or less education in university regarding eHealth	We don't really have contact with digitized systems... we don't learn about them
Reasons: Disagree / Strongly disagree\there is no or less education in university regarding eHealth	As eHealth was lacking from my medical education, I would need training before using any eHealth in the clinical field.
Reasons: Disagree / Strongly disagree\there is no or less education in university regarding eHealth	my faculty hasn't prepared me to work in any system at all
Reasons: Disagree / Strongly disagree\there is no or less education in university regarding eHealth	Not nearly enough attention is paid to the machines and technology behind medicine. Mainly to learn the theory to practise medicine without needing them, but they will become common.
Reasons: Disagree / Strongly disagree\there is no or less education in university regarding eHealth	No knowledge about this aspect of healthcare is being given to us, albeit we'll welcome it open heartedly.
Reasons: Disagree / Strongly disagree\there is no or less education in university regarding eHealth	3rd year student, I don’t know what is yet to come.
Reasons: Disagree / Strongly disagree\there is no or less education in university regarding eHealth	not yet in the clinical curriculum
Reasons: Disagree / Strongly disagree\there is no or less education in university regarding eHealth	My ability to use electronic devices and software in general is good, still it would be nice to be prepared for specific software and situations. In university digitization is not considered at all, neither in teaching nor in patient care.
Reasons: Disagree / Strongly disagree\there is no or less education in university regarding eHealth	I don't feel prepared because I didn't recieve any training on the matter yet
Reasons: Disagree / Strongly disagree\no/not enough experiences/practice so far	I don't have any experience with it.
Reasons: Disagree / Strongly disagree\no/not enough experiences/practice so far	It's actually the first time I hear something about e-health, how could I fell prepared for working through it?
Reasons: Disagree / Strongly disagree\no/not enough experiences/practice so far	I'm not experienced enough with healthcare devices to consider myself literate.
Reasons: Disagree / Strongly disagree\no/not enough experiences/practice so far	Lack of training
Reasons: Disagree / Strongly disagree\no/not enough experiences/practice so far	I have nearly no experience.

Reasons: Disagree / Strongly disagree\no/not enough experiences/practice so far	I haven't received enough eHealth knowledge or practical skills in order to be able to work appropriately in a digitized healthcare system.
Reasons: Disagree / Strongly disagree\no/not enough experiences/practice so far	We haven't yet practiced anything regarding ehealth.
Reasons: Disagree / Strongly disagree\no/not enough experiences/practice so far	"Ubung macht den Meister" as the German say. Without practice I will lead my self to a black hole.
Reasons: Disagree / Strongly disagree\no/not enough experiences/practice so far	training
Reasons: Disagree / Strongly disagree\no/not enough experiences/practice so far	I did not attend training yet.
Reasons: Disagree / Strongly disagree\no/not enough experiences/practice so far	have
Reasons: Disagree / Strongly disagree\no/not enough experiences/practice so far	im just finding out about e-health
Reasons: Disagree / Strongly disagree\no/not enough experiences/practice so far	As eHealth was lacking from my medical education, I would need training before using any eHealth in the clinical field.
Reasons: Disagree / Strongly disagree\no/not enough experiences/practice so far	I'm still early in my medical career and hope to develop these skills
Reasons: Disagree / Strongly disagree\no/not enough experiences/practice so far	I havent been exposed to much information regarding ehealth before this survey. So i believe i am not prepared and would love to get more knowledge on this topic
Reasons: Disagree / Strongly disagree\no/not enough experiences/practice so far	I was never trained to do this
Reasons: Disagree / Strongly disagree\no/not enough experiences/practice so far	I haven't been professionally prepared in that regard
Reasons: Disagree / Strongly disagree\no/not enough experiences/practice so far	I dont have enough experience to answer that
Reasons: Disagree / Strongly disagree\no/not enough experiences/practice so far	I don't feel prepared because I didn't recieve any training on the matter yet
