# Supplementary material for: Perceptions of Digital Health Education Among European Medical Students: Mixed Methods Survey
Source: J Med Internet Res. 2020 Aug 14;22(8):e19827. doi: 10.2196/19827 (PMC7455864; doi:10.2196/19827)
Supplement: Multimedia Appendix 5 [file jmir_v22i8e19827_app5.pdf]

What specific eHealth-related courses would you like to have in your university's curriculum?

Color	Code	Segment
●	implementation into medical education\what?\Topics\training with technologies	as well as courses on how to use already existing eHealth services that are being used at the university clinic.
●	implementation into medical education\what?\Topics\training with technologies	Usage of eHealth technologies
●	implementation into medical education\what?\Topics\training with technologies	Using new technologys
●	implementation into medical education\what?\Topics\training with technologies	How to use it
●	implementation into medical education\what?\Topics\training with technologies	eHealth usage
●	implementation into medical education\what?\Topics\training with technologies	different computer programs available and how to use them for a better medical act, etc
●	implementation into medical education\what?\Topics\training with technologies	Ehealth apps exploring, together with medical specialists and IT guides. Courses on diagnostic tools available for doctors. A course where you explore free open source apps or programs that monitor patients, a lot on big data analysis for health regarding research & how to read papers on big data analysis that are done
●	implementation into medical education\what?\Topics\training with technologies	Programming, usage of the most common eHealth Tools
●	implementation into medical education\what?\Topics\training with technologies	How to use smartphones in diagnostics and how to collect big data from there
●	implementation into medical education\what?\Topics\training with technologies	Practical apps
●	implementation into medical education\what?\Topics\training with technologies	How to use eHealth technologies, information about different apps etc
●	implementation into medical education\what?\Topics\training with technologies	Usage of ehealth technologies
●	implementation into medical education\what?\Topics\training with technologies	How we can use it in our daily practice as doctors, how it will effect the doctor - patient relationship and what ethical dillemas it brings
●	implementation into medical education\what?\Topics\training with technologies	How to develop ie use
●	implementation into medical education\what?\Topics\training with technologies	research and usage of instruments in clinical practice

●	implementation into medical education\what?\Topics\training with technologies	practical
●	implementation into medical education\what?\Topics\training with technologies	usage of health apps; courses on teaching patients how to deal with those apps, what the benefits and dangers are.
●	implementation into medical education\what?\Topics\training with technologies	Training to use it
●	implementation into medical education\what?\Topics\training with technologies	Common technologies and their application
●	implementation into medical education\what?\Topics\training with technologies	get familiar with the apps
●	implementation into medical education\what?\Topics\training with technologies	How to use eHealth, programming
●	implementation into medical education\what?\Topics\training with technologies	Programming and use of the e-instruments.
●	implementation into medical education\what?\Topics\training with technologies	If some ehealth solutions would be implemented into day to day patient care I would like to have courses on how to use them
●	implementation into medical education\what?\Topics\training with technologies	I would really like to get the courses about diagnosis over electronic devices like cell-phones. I would like to know the criteria while examining a patient and see the database of it.
●	implementation into medical education\what?\Topics\training with technologies	- How to use eHealth services and improve medical decisions
●	implementation into medical education\what?\Topics\training with technologies	They should teach us how the given program works, how can be teach the usage of these to our future patients, introducing to the newest technologies
●	implementation into medical education\what?\Topics\training with technologies	available technologies and their usage
●	implementation into medical education\what?\Topics\training with technologies	Training in and knowledge of the current existing options. A look at what options are coming in the near future.
●	implementation into medical education\what?\Topics\training with technologies	Practical course about how to use telematic systems
●	implementation into medical education\what?\Topics\training with technologies	More in exercises
●	implementation into medical education\what?\Topics\training with technologies	How to use programs
●	implementation into medical education\what?\Topics\training with technologies	How to use x

●	implementation into medical education\what?\Topics\training with technologies	Use of patient record systems and safety
●	implementation into medical education\what?\Topics\training with technologies	updates on current programms/usages
●	implementation into medical education\what?\Topics\training with technologies	course on ehtical question related to eHealth, course on how to use apps/programms that I'll actually use in my future work life
●	implementation into medical education\what?\Topics\training with technologies	I would like to learn specifically and practically about the use of e-descriptions and the systems about remote control machines.
●	implementation into medical education\what?\Topics\training with technologies	The use of eHealth and specifically mHealth and TeleHealth in medicine, Big Data usage, Correct usage of eHealth technologies serving the patient's best interests.
●	implementation into medical education\what?\Topics\training with technologies	How to use eHealth in treatment of patients
●	implementation into medical education\what?\Topics\training with technologies	One that could provide the students with the use of new technologies in medicine.
●	implementation into medical education\what?\Topics\training with technologies	Patient data handling and safety, e-healthcare system (in Hungary we just implemented a national system that has all patient records/e-prescriptions, etc that is available for doctors + to dentists + to pharmacists, but we as medical students receive no training on it whatsoever)
●	implementation into medical education\what?\Topics\training with technologies	practical approach : Whic Technology which specific purpose.
●	implementation into medical education\what?\Topics\training with technologies	Training upon usage of technology at health care (electronic prescription, sharing informations). Discusssion upon ethical limitations of sharing patients' information. Training for Telemedicine (2 groups, one being the patient and the other the medical team, discussing how to approach and decrease the distance between them).
●	implementation into medical education\what?\Topics\training with technologies	Ethical discurs, training in programming
●	implementation into medical education\what?\Topics\training with technologies	Ethische Diskussionen, Darstellung der Möglichkeiten, praktische Übungen
●	implementation into medical education\what?\Topics\training with technologies	app usage, medical registry,
●	implementation into medical education\what?\Topics\training with technologies	Programming applied to health, highlighting theory and applications behind common examples

●	implementation into medical education\what?\Topics\training with technologies	How to handle data, how to ensure privacy of the patients, how to manage health apps and incorporate them in your daily practice.
●	implementation into medical education\what?\Topics\training with technologies	practice/related in the different fields as well as the opportunity to use programming to improve on/understand the data science behind it
●	implementation into medical education\what?\Topics\training with technologies	E health application
●	implementation into medical education\what?\Topics\training with technologies	Usage of eHealth technologies
●	implementation into medical education\what?\Topics\training with technologies	eHealth Skills and how to deal with wearables
●	implementation into medical education\what?\Topics\training with technologies	Workshops and exercises in more disciplines (teachers should explain that the exercises are related with eHealth knowledge).
●	implementation into medical education\what?\Topics\training with technologies	Use of medical record keeping in practice
●	implementation into medical education\what?\Topics\training with technologies	Using apps about medical knowledge
●	implementation into medical education\what?\Topics\training with technologies	Problem Oriented training
●	implementation into medical education\what?\Topics\statistics	statistical evaluation
●	implementation into medical education\what?\Topics\statistics	Stastitical packages
●	implementation into medical education\what?\Topics\statistics	Statistics
●	implementation into medical education\what?\Topics\Research Opportunities	Research opportunities
●	implementation into medical education\what?\Topics\Research Opportunities	Programming languages, app designing, presentation of health start-ups, dealing with data and (legal) restrictions with that, computer science, research opportunities
●	implementation into medical education\what?\Topics\Research Opportunities	research and usage of instruments in clinical practice
●	implementation into medical education\what?\Topics\Research Opportunities	Research courses
●	implementation into medical education\what?\Topics\Research Opportunities	Courses on big data, analytics, and how this relates to research and clinical decision making. (Definitely after pre-med. Maybe in the 4th o 5th year.)

●	implementation into medical education\what?\Topics\Research Opportunities	Using of big data, research skills.
●	implementation into medical education\what?\Topics\Research Opportunities	Research
●	implementation into medical education\what?\Topics\Research Opportunities	Need more research
●	implementation into medical education\what?\Topics\Research Opportunities	I would like to learn about its possibilities in research
●	implementation into medical education\what?\Topics\Research Opportunities	data management; working with CDSS; research; computer science
●	implementation into medical education\what?\Topics\Research Opportunities	Research
●	implementation into medical education\what?\Topics\Research Opportunities	different forms and usages of eHealth in general patient care; advantages and risks, background information on the collection and protection of data, ways on how to use the gathered data for research.
●	implementation into medical education\what?\Topics\precision medicine	individual Patient treatment
●	implementation into medical education\what?\Topics\precision medicine	Introduction on e-health era, ECG, MRI, Measuring the levels of glucose and the heartbeat, Advice on personalized medicine, Privacy and protecting the patients from new diseases and instant and sudden health problems.
●	implementation into medical education\what?\Topics\precision medicine	Clinical decision support, machine learning, precision medicine.
●	implementation into medical education\what?\Topics\operating principles of technologies	They should teach us how the given program works, how can be teach the usage of these to our future patients, introducing to the newest technologies
●	implementation into medical education\what?\Topics\operating principles of technologies	Course on how different eHealth apps work, where you can "meet" a doctor in n app instead of physically going a healt care facility. Many patients have consulted apps when they're unwell and it would be useful to learn about what kind of help you can expect they have received.
●	implementation into medical education\what?\Topics\operating principles of technologies	the processes involved in eHealth
●	implementation into medical education\what?\Topics\operating principles of technologies	Telecare, E health policy framework, E health devices principles

	implementation into medical education\what?\Topics\operating principles of technologies	Electronic Health insurance card, Electronic Health Records, how Health Apps work, CDSS, (telemedicine)
	implementation into medical education\what?\Topics\operating principles of technologies	Information about diagnostic-helpers
	implementation into medical education\what?\Topics\legal issues	Programming languages, app designing, presentation of health start-ups, dealing with data and (legal) restrictions with that, computer science, research opportunities
	implementation into medical education\what?\Topics\legal issues	A general overview of the advantages and disadvantages of such a system. Possible uses. Regulations. Ethics.
	implementation into medical education\what?\Topics\legal issues	statistics, what is possible by law,
	implementation into medical education\what?\Topics\legal issues	Telecare, E health policy framework, E health devices principles
	implementation into medical education\what?\Topics\legal issues	legal
	implementation into medical education\what?\Topics\Informatics\programming languages \app development	Statistical data analysing, app development
	implementation into medical education\what?\Topics\Informatics\programming languages \app development	I'd like to attend a classes where we can learn all the basics and an optional one if someone'd like to develope or upgarde the exsisting opportonuties.
	implementation into medical education\what?\Topics\Informatics\programming languages \app development	To learn how to program new mobile apps
	implementation into medical education\what?\Topics\Informatics\programming languages \app development	I would love to have a lesson about programming (for example some basic information about how to develop an app)
	implementation into medical education\what?\Topics\Informatics\programming languages	On the use of big data and the ethical reasons underlying that, on programming languages that are very useful in pursuing a career in research and on the use of AI in medicine.
	implementation into medical education\what?\Topics\Informatics\programming languages	Research programs, bigdata,
	implementation into medical education\what?\Topics\Informatics\programming languages	Structured introduction into statistic programs and programming

•	implementation into medical education\what?\Topics\Informatics\programming languages	Long distance monitoring softwares and related coding languages
•	implementation into medical education\what?\Topics\Informatics\programming languages	programming
•	implementation into medical education\what?\Topics\Informatics\programming languages	Programming
•	implementation into medical education\what?\Topics\Informatics\programming languages	Program languages
•	implementation into medical education\what?\Topics\Informatics\programming languages	Programming languages, app designing, presentation of health start-ups, dealing with data and (legal) restrictions with that, computer science, research opportunities
•	implementation into medical education\what?\Topics\Informatics\programming languages	programming
•	implementation into medical education\what?\Topics\Informatics\programming languages	eHealth 101 and a programming language course.
•	implementation into medical education\what?\Topics\Informatics\programming languages	How to use eHealth, programming
•	implementation into medical education\what?\Topics\Informatics\programming languages	Programming and use of the e-instruments.
•	implementation into medical education\what?\Topics\Informatics\programming languages	Programming, AI, data science (other than stats)
•	implementation into medical education\what?\Topics\Informatics\programming languages	To learn how to program new mobile apps
•	implementation into medical education\what?\Topics\Informatics\programming languages	Programming courses especially concerning data science, introduction to machine learning with focus on image recognition (applicable for example in radiological contexts)

•	implementation into medical education\what?\Topics\Informatics\programming languages	Artificial intelligence courses, Ethical issues, Programming courses
•	implementation into medical education\what?\Topics\Informatics\programming languages	programming courses
•	implementation into medical education\what?\Topics\Informatics\programming languages	Information on mobile apps, wearables and telemedicine, artificial intelligence, programming skills, software engineering
•	implementation into medical education\what?\Topics\Informatics\programming languages	basic programming could be useful to some.
•	implementation into medical education\what?\Topics\Informatics\programming languages	Familiarization with electronic prescriptions, telemedicine courses and programming languages.
•	implementation into medical education\what?\Topics\Informatics\programming languages	coding and data usage
•	implementation into medical education\what?\Topics\Informatics\programming languages	Ethical discussions, basic programming knowledge, examples of opportunities and already existing tools ...
•	implementation into medical education\what?\Topics\Informatics\programming languages	Pros and cons and the basics of programming
•	implementation into medical education\what?\Topics\Informatics\programming languages	Programming applied to health, highlighting theory and applications behind common examples
•	implementation into medical education\what?\Topics\Informatics\programming languages	Classes about big data and how it can be put in practice. Programming. Medical Software
•	implementation into medical education\what?\Topics\Informatics\programming languages	programming
•	implementation into medical education\what?\Topics\Informatics\programming languages	programming, more technical subjects and interships
•	implementation into medical education\what?\Topics\Informatics\programming languages	data management; working with CDSS; research; computer science

●	implementation into medical education\what?\Topics\Informatics\basic principles of interoperability	Interoperability at the grassrouts level
●	implementation into medical education\what?\Topics\Informatics	Infoematics
●	implementation into medical education\what?\Topics\Informatics	More research classes,medical informatics classes
●	implementation into medical education\what?\Topics\Informatics	how to create software adapted to the way I work, how to protect my patients data
●	implementation into medical education\what?\Topics\Informatics	computer science, telemedicine
●	implementation into medical education\what?\Topics\Informatics	Medical Informatics (Health Data Science)
●	implementation into medical education\what?\Topics\Informatics	general IT knowledge, how to handle CDSS
●	implementation into medical education\what?\Topics\Informatics	AI, computer science, big data.
●	implementation into medical education\what?\Topics\Informatics	Understanding of IT dynamics
●	implementation into medical education\what?\Topics\Informatics	Computer skills
●	implementation into medical education\what?\Topics\Informatics	Bio medical and ehealth informatics
●	implementation into medical education\what?\Topics\Informatics	word programming and CDCC
●	implementation into medical education\what?\Topics\ethical aspects	On the use of big data and the ethical reasons underlying that
●	implementation into medical education\what?\Topics\ethical aspects	eHealth and doctors (to explore more about the ethical and the everyday issues that come up from the integration of eHealth in the system)
●	implementation into medical education\what?\Topics\ethical aspects	as well as more info and discussions on the ethic questions surrounding this matter.
●	implementation into medical education\what?\Topics\ethical aspects	ethical discussions
●	implementation into medical education\what?\Topics\ethical aspects	ethical discussion
●	implementation into medical education\what?\Topics\ethical aspects	Seminar on Ethical Aspects of eHealth and access to health data

●	implementation into medical education\what?\Topics\ethical aspects	ethical discussions, which advantages and risks?
●	implementation into medical education\what?\Topics\ethical aspects	Ethical discussions about big data
●	implementation into medical education\what?\Topics\ethical aspects	ethical implications
●	implementation into medical education\what?\Topics\ethical aspects	How we can use it in our daily practice as doctors, how it will effect the doctor - patient relationship and what ethical dillemas it brings
●	implementation into medical education\what?\Topics\ethical aspects	Soft skill Trainings, ethical discussions
●	implementation into medical education\what?\Topics\ethical aspects	ethical implications of eHealth and big data
●	implementation into medical education\what?\Topics\ethical aspects	A general overview of the advantages and disadvantages of such a system. Possible uses. Regulations. Ethics.
●	implementation into medical education\what?\Topics\ethical aspects	Start-up opportunities, ethical aspects of ehealth.
●	implementation into medical education\what?\Topics\ethical aspects	presentation of different already implemented apps/Tools, discussion about use/harm, discussion about how to use it to our Advantage as future medical staff
●	implementation into medical education\what?\Topics\ethical aspects	Ethical discussion perhaps
●	implementation into medical education\what?\Topics\ethical aspects	Artificial intelligence courses, Ethical issues, Programming courses
●	implementation into medical education\what?\Topics\ethical aspects	course on ehtical question related to eHealth, course on how to use apps/programms that I'll actually use in my future work life
●	implementation into medical education\what?\Topics\ethical aspects	use of basic e-health methods and tools, the ethics and the barriers that we should apply to e-health usage
●	implementation into medical education\what?\Topics\ethical aspects	Training upon usage of technology at health care (electronic prescription, sharing informations). Discusssion upon ethical limitations of sharing patients' information. Training for Telemedicine (2 groups, one being the patient and the other the medical team, discussing how to approach and decrease the distance between them).
●	implementation into medical education\what?\Topics\ethical aspects	Ethical discurs, training in programming
●	implementation into medical education\what?\Topics\ethical aspects	Ethische Diskussionen, Darstellung der Möglichkeiten, praktische Übungen

●	implementation into medical education\what?\Topics\ethical aspects	Ethical discussions, basic programming knowledge, examples of opportunities and already existing tools ...
●	implementation into medical education\what?\Topics\ethical aspects	EHealth, Big Data and ethics as a core course
●	implementation into medical education\what?\Topics\ethical aspects	Ethical discussion
●	implementation into medical education\what?\Topics\ethical aspects	More information and discussions on big data and how for example pharmaceutical companies use big data to their advantage
●	implementation into medical education\what?\Topics\ethical aspects	eHealth awareness
●	implementation into medical education\what?\Topics\enterpreneurship	How to develop an App related to eHealth (maybe by having representatives from certain start-up companies come to universities),
●	implementation into medical education\what?\Topics\enterpreneurship	How to develop ie use
●	implementation into medical education\what?\Topics\enterpreneurship	Programming languages, app designing, presentation of health start-ups, dealing with data and (legal) restrictions with that, computer science, research opportunities
●	implementation into medical education\what?\Topics\enterpreneurship	Start-up opportunities, ethical aspects of ehealth.
●	implementation into medical education\what?\Topics\enterpreneurship	startup development, data managing, statistical analysis
●	implementation into medical education\what?\Topics\enterpreneurship	About decision making and start-up opportunities
●	implementation into medical education\what?\Topics\eHealth in health systems\status of digitization in healthcare in own country	Situation in my country.
●	implementation into medical education\what?\Topics\eHealth in health systems\status of digitization in healthcare in own country	Comparisons of different countries approaches to implementing healthcare ICT and barriers in our system
●	implementation into medical education\what?\Topics\eHealth in health systems\how to: implementation of eHealth	Courses on how to implement eHealth in clinical practice
●	implementation into medical education\what?\Topics\eHealth in health systems\how to: implementation of eHealth	How to use these new technologies for our formation and patients

●	implementation into medical education\what?\Topics\eHealth in health systems\how to: implementation of eHealth	Comparisons of different countries approaches to implementing healthcare ICT and barriers in our system
●	implementation into medical education\what?\Topics\eHealth in health systems\how to: implementation of eHealth	Wise use of new eHealth services, implementation, risks and benefits, "how to addapt" (for patients and as a future doc)
●	implementation into medical education\what?\Topics\Data Management\Data sharing	Data sharing
●	implementation into medical education\what?\Topics\Data Management\Data Security	information about security of patient data
●	implementation into medical education\what?\Topics\Data Management\Data Security	d safety of using patient data
●	implementation into medical education\what?\Topics\Data Management\Data Security	Security and privacy on eHealth, Records on eHealth
●	implementation into medical education\what?\Topics\Data Management\Data Security	how to create software adapted to the way I work, how to protect my patients data
●	implementation into medical education\what?\Topics\Data Management\Data Security	Protection of patients data
●	implementation into medical education\what?\Topics\Data Management\Data Security	Security, haw to use it, what to say to patients
●	implementation into medical education\what?\Topics\Data Management\Data Security	Use of patient record systems and safety
●	implementation into medical education\what?\Topics\Data Management\Data Security	secure data
●	implementation into medical education\what?\Topics\Data Management\Data Security	Introduction on e-health era, ECG, MRI, Measuring the levels of glucose and the heartbeat, Advice on personalized medicine, Privacy and protecting the patients from new diseases and instant and sudden health problems.
●	implementation into medical education\what?\Topics\Data Management\Data Security	How to handle data, how to ensure privacy of the patients, how to manage health apps and incorporate them in your daily practice.
●	implementation into medical education\what?\Topics\Data Management\Big Data	Big Data
●	implementation into medical education\what?\Topics\Data Management\Big Data	On the use of big data
●	implementation into medical education\what?\Topics\Data Management\Big Data	Big data, Patient monitoring
●	implementation into medical education\what?\Topics\Data Management\Big Data	Big Data

●	implementation into medical education\what?\Topics\Data Management\Big Data	Ethical discussions about big data
●	implementation into medical education\what?\Topics\Data Management\Big Data	a lot on big data analysis for health regarding research & how to read papers on big data analysis that are don
●	implementation into medical education\what?\Topics\Data Management\Big Data	Use of big data in research,
●	implementation into medical education\what?\Topics\Data Management\Big Data	mHealth oppurtunities. Courses on latest eHealth implementations. More courses on Big Data systems in use at the moment.
●	implementation into medical education\what?\Topics\Data Management\Big Data	Usage of big data
●	implementation into medical education\what?\Topics\Data Management\Big Data	Big data
●	implementation into medical education\what?\Topics\Data Management\Big Data	(apps, data systems
●	implementation into medical education\what?\Topics\Data Management\Big Data	Courses on big data, analytics, and how this relates to research and clinical decision making. (Definitely after pre-med. Maybe in the 4th o 5th year.)
●	implementation into medical education\what?\Topics\Data Management\Big Data	Using of big data, research skills.
●	implementation into medical education\what?\Topics\Data Management\Big Data	learning about documenting health information in a way that allows us to participate in contributing to big data if we choose to
●	implementation into medical education\what?\Topics\Data Management\Big Data	Dealing with big data
●	implementation into medical education\what?\Topics\Data Management\Big Data	Use of big data, artificial intelligence, decision making tools
●	implementation into medical education\what?\Topics\Data Management\Big Data	AI, computer science, big data.
●	implementation into medical education\what?\Topics\Data Management\Big Data	The use of eHealth and specifically mHealth and TeleHealth in medicine, Big Data usage, Correct usage of eHealth technologies serving the patient's best interests.
●	implementation into medical education\what?\Topics\Data Management\Big Data	Modern developments in medical Artficial Intelligence, Analysis of Big Data
●	implementation into medical education\what?\Topics\Data Management\Big Data	Big Data
●	implementation into medical education\what?\Topics\Data Management\Big Data	Artificial Intelligence and Big Data

•	implementation into medical education\what?\Topics\Data Management\Big Data	Overview, teleHealth, Big Data
•	implementation into medical education\what?\Topics\Data Management\Big Data	Use of Big Data.
•	implementation into medical education\what?\Topics\Data Management\Big Data	Big data
•	implementation into medical education\what?\Topics\Data Management\Big Data	Big data, telemedicine
•	implementation into medical education\what?\Topics\Data Management\Big Data	Big data use
•	implementation into medical education\what?\Topics\Data Management\Big Data	Big data
•	implementation into medical education\what?\Topics\Data Management\Big Data	Classes about big data and how it can be put in practice. Programming. Medical Software
•	implementation into medical education\what?\Topics\Data Management\Big Data	EHealth, Big Data and ethics as a core course
•	implementation into medical education\what?\Topics\Data Management\Big Data	Learning how to access big data sources, other sources of information.
•	implementation into medical education\what?\Topics\Data Management\Big Data	Big data, clinical apps
•	implementation into medical education\what?\Topics\Data Management\Big Data	More information and discussions on big data and how for example pharmaceutical companies use big data to their advantage
•	implementation into medical education\what?\Topics\Data Management	data management
•	implementation into medical education\what?\Topics\Data Management	How to handle electronic health data efficiently,
•	implementation into medical education\what?\Topics\Data Management	data analyze
•	implementation into medical education\what?\Topics\Data Management	better data-science classes
•	implementation into medical education\what?\Topics\Data Management	how the programs get their data
•	implementation into medical education\what?\Topics\Data Management	How to use smartphones in diagnostics and how to collect big data from there
•	implementation into medical education\what?\Topics\Data Management	Database management

•	implementation into medical education\what?\Topics\Data Management	Usage of visuals, artificial intellegience, virtual reality, more databases and revisioned and appopriate databases for current lessions
•	implementation into medical education\what?\Topics\Data Management	Programming languages, app designing, presentation of health start-ups, dealing with data and (legal) restrictions with that, computer science, research opportunities
•	implementation into medical education\what?\Topics\Data Management	About data bases, telemedicine
•	implementation into medical education\what?\Topics\Data Management	Data management, monitoring, mobile applications
•	implementation into medical education\what?\Topics\Data Management	Clinical database training, Patient record retrieval training,
•	implementation into medical education\what?\Topics\Data Management	Statistical data analysing, app development
•	implementation into medical education\what?\Topics\Data Management	Info about mobile apps, learning about their pros and cons, so we can help patients, which one to use. We should also learn to use big data systems, we should be able to search in databases.
•	implementation into medical education\what?\Topics\Data Management	Programming, AI, data science (other than stats)
•	implementation into medical education\what?\Topics\Data Management	Working with data (statistically, analitically...)
•	implementation into medical education\what?\Topics\Data Management	Data managing and online programmes
•	implementation into medical education\what?\Topics\Data Management	Programming courses especially concerning data science, introduction to machine learning with focus on image recognition (applicable for example in radiological contexts)
•	implementation into medical education\what?\Topics\Data Management	Artificial intelligence, patient data programms (SAP?)
•	implementation into medical education\what?\Topics\Data Management	More Data Science
•	implementation into medical education\what?\Topics\Data Management	Medical Informatics (Health Data Science)
•	implementation into medical education\what?\Topics\Data Management	startup development, data managing, statistical analysis
•	implementation into medical education\what?\Topics\Data Management	Usage of online libraries, excel data sheet

	implementation into medical education\what?\Topics\Data Management	Artificial intelligence programs, clinical trial data handling
	implementation into medical education\what?\Topics\Data Management	critical and responsible use of data, data security, broadening the horizon of possible applications and possibilities
	implementation into medical education\what?\Topics\Data Management	Patient data handling and safety, e-healthcare system (in Hungary we just implemented a national system that has all patient records/e-prescriptions, etc that is available for doctors + to dentists + to pharmacists, but we as medical students receive no training on it whatsoever)
	implementation into medical education\what?\Topics\Data Management	usage of data (how to), telemedicine
	implementation into medical education\what?\Topics\Data Management	coding and data usage
	implementation into medical education\what?\Topics\Data Management	How to handle data, how to ensure privacy of the patients, how to manage health apps and incorporate them in your daily practice.
	implementation into medical education\what?\Topics\Data Management	practice/related in the different fields as well as the opportunity to use programming to improve on/understand the data science behind it
	implementation into medical education\what?\Topics\Data Management	Using the recorded data for diagnosis and therapy
	implementation into medical education\what?\Topics\Data Management	data management; working with CDSS; research; computer science
	implementation into medical education\what?\Topics\Data Management	different forms and usages of eHealth in general patient care; advantages and risks, background information on the collection and protection of data, ways on how to use the gathered data for research.
	implementation into medical education\what?\Topics\communication skills\how to deal with the "e-Patient"\educating patients (digital health literacy)	New technologies, how to use them. Patient education
	implementation into medical education\what?\Topics\communication skills\how to deal with the "e-Patient"\educating patients (digital health literacy)	usage of health apps; courses on teaching patients how to deal with those apps, what the benefits and dangers are.

●	implementation into medical education\what?\Topics\communication skills\how to deal with the "e-Patient"\educating patients (digital health literacy)	Prevention
●	implementation into medical education\what?\Topics\communication skills\how to deal with the "e-Patient"\educating patients (digital health literacy)	More examples of how it could be used and more infos about good sites we could redirect our patients to
●	implementation into medical education\what?\Topics\communication skills\how to deal with the "e-Patient"\educating patients (digital health literacy)	E Health literacy
●	implementation into medical education\what?\Topics\communication skills\how to deal with the "e-Patient"\educating patients (digital health literacy)	Wise use of new eHealth services, implementation, risks and benefits, "how to addapt" (for patients and as a future doc)
●	implementation into medical education\what?\Topics\communication skills\how to deal with the "e-Patient"	New technology and dealing with patients that already use eHealth by themselves
●	implementation into medical education\what?\Topics\communication skills\how to deal with the "e-Patient"	How we can use it in our daily practice as doctors, how it will effect the doctor - patient relationship and what ethical dillemas it brings
●	implementation into medical education\what?\Topics\communication skills\how to deal with the "e-Patient"	Soft skill Trainings, ethical discussions
●	implementation into medical education\what?\Topics\communication skills\how to deal with the "e-Patient"	Info about mobile apps, learning about their pros and cons, so we can help patients, which one to use. We should also learn to use big data systems, we should be able to search in databases.
●	implementation into medical education\what?\Topics\communication skills\how to deal with the "e-Patient"	At least a basic understand of what is available to patients and how we can help implement eHealth into patient care as well as help with any queries patients may have about it.
●	implementation into medical education\what?\Topics\communication skills\how to deal with the "e-Patient"	eHealth and patients
●	implementation into medical education\what?\Topics\communication skills\how to deal with the "e-Patient"	Security, haw to use it, what to say to patients

●	implementation into medical education\what?\Topics\communication skills\how to deal with the "e-Patient"	Course on how different eHealth apps work, where you can "meet" a doctor in n app instead of physically going a healt care facility. Many patients have consulted apps when they're unwell and it would be useful to learn about what kind of help you can expect they have received.
●	implementation into medical education\what?\Topics\communication skills\how to deal with the "e-Patient"	Apps for patients, support and knwlesge systems for doctors
●	implementation into medical education\what?\Topics\communication skills\course to decrease animosity	Since we got none, basic principles and sth to reduce the animosity.
●	implementation into medical education\what?\Topics	Learn how to manipulate
●	implementation into medical education\what?\Topics	Biological imaging
●	implementation into medical education\what?\Topics	which infocommunications
●	implementation into medical education\what?\overview/basics	Basic knowledge,
●	implementation into medical education\what?\overview/basics	A basics seminar
●	implementation into medical education\what?\overview/basics	Overviews
●	implementation into medical education\what?\overview/basics	Different options and their advantages and disadvantages,
●	implementation into medical education\what?\overview/basics	General introduction
●	implementation into medical education\what?\overview/basics	Literacy
●	implementation into medical education\what?\overview/basics	Risks and Profits. General Introduktion
●	implementation into medical education\what?\overview/basics	Overview
●	implementation into medical education\what?\overview/basics	Recent technological advances and implementations of eHealth in every domain of medicine.
●	implementation into medical education\what?\overview/basics	Courses which are showing me at least the current possibilities and the future opportunities of ehealth.
●	implementation into medical education\what?\overview/basics	An overview with examples.
●	implementation into medical education\what?\overview/basics	General outline of e-Health

●	implementation into medical education\what?\overview/basics	Overview over technologies and possible advantages as well as risks
●	implementation into medical education\what?\overview/basics	a general talk on how to use ehealth to improve patients health.
●	implementation into medical education\what?\overview/basics	Well, every course if possible. We are young professionals, so it’s save to assume that we will all depend on ehealth and that ehealth will only get bigger
●	implementation into medical education\what?\overview/basics	Since we got none, basic principles and sth to reduce the animosity.
●	implementation into medical education\what?\overview/basics	eHealth 101 and a programming language course.
●	implementation into medical education\what?\overview/basics	Since I don't have courses at all, a basic introductive course could be ok
●	implementation into medical education\what?\overview/basics	General eHealth and its uses
●	implementation into medical education\what?\overview/basics	an introduction to the different branches and their use in our career
●	implementation into medical education\what?\overview/basics	A general overview of the advantages and disadvantages of such a system. Possible uses. Regulations. Ethics.
●	implementation into medical education\what?\overview/basics	A bit of everything
●	implementation into medical education\what?\overview/basics	At least a basic understand of what is available to patients and how we can help implement eHealth into patient care as well as help with any queries patients may have about it.
●	implementation into medical education\what?\overview/basics	I'd like to attend a classes where we can learn all the basics and an optional one if someone'd like to develope or upgarde the exsisting opportonuties.
●	implementation into medical education\what?\overview/basics	presentation of different already implemented apps/Tools, discussion about use/harm, discussion about how to use it to our Advantage as future medical staff<br>In General mainly just to educate about it, and not ignoring it in our Curriculum as if it does not exist
●	implementation into medical education\what?\overview/basics	A general overview with the possibility to look into specific topics in detail
●	implementation into medical education\what?\overview/basics	not yet sensefull, but in future will be more necessary to give an overview to all possabilities
●	implementation into medical education\what?\overview/basics	More about awareness on eHealth and how we can make this usefull in our future

●	implementation into medical education\what?\overview/basics	something general about eHealth
●	implementation into medical education\what?\overview/basics	Just the basics of what to expect later on in my job.
●	implementation into medical education\what?\overview/basics	critical and responsible use of data, data security, broadening the horizon of possible applications and possibilities
●	implementation into medical education\what?\overview/basics	general
●	implementation into medical education\what?\overview/basics	Wise use of new eHealth services, implementation, risks and benefits, "how to addapt" (for patients and as a future doc)
●	implementation into medical education\what?\overview/basics	use of basic e-health methods and tools, the ethics and the barriers that we should apply to e-health usage
●	implementation into medical education\what?\overview/basics	eHealth system 101
●	implementation into medical education\what?\overview/basics	up-to-date awareness about fundamental eHealth apps and/ or devices
●	implementation into medical education\what?\overview/basics	Introduction on e-health era, ECG, MRI, Measuring the levels of glucose and the heartbeat, Advice on personalized medicine, Privacy and protecting the patients from new diseases and instant and sudden health problems.
●	implementation into medical education\what?\overview/basics	Current and future trends in mHealth and eHealth solutions
●	implementation into medical education\what?\overview/basics	E-health basics
●	implementation into medical education\what?\overview/basics	Basics
●	implementation into medical education\what?\overview/basics	Overview, teleHealth, Big Data
●	implementation into medical education\what?\overview/basics	Ethical discussions, basic programming knowledge, examples of opportunities and already existing tools ...
●	implementation into medical education\what?\overview/basics	I believe at least a lecture highlighting the most important aspects when it comes to eHealth is necessary and could easily be implemented.
●	implementation into medical education\what?\overview/basics	I'm not even sure because I feel I don't know what eHealth truly represents. So every possible course, starting with a small one about technology in Medicine.

●	implementation into medical education\what?\overview/basics	Basic stuff
●	implementation into medical education\what?\overview/basics	Pros and cons and the basics of programming
●	implementation into medical education\what?\overview/basics	EHealth, Big Data and ethics as a core course
●	implementation into medical education\what?\overview/basics	explaining better what eHealth is and how it makes us work better
●	implementation into medical education\what?\overview/basics	different forms and usages of eHealth in general patient care; advantages and risks, background information on the collection and protection of data, ways on how to use the gathered data for research.
●	implementation into medical education\what?\overview/basics	eHealth basics in general
●	implementation into medical education\what?\overview/basics	eHealth literacy
●	implementation into medical education\what?\overview/basics	I want to have a ehealth literacy. Big data, AI seems to be too scientific for doctor, but in general it would be great to understand what and how it works and why it is needed.
●	implementation into medical education\what?\overview/basics	Anything to start with
●	implementation into medical education\what?\overview/basics	I don't know enough about the topic to name any courses. General knowledge about eHealth would be nice
●	implementation into medical education\what?\overview/basics	One that would teach me how to participe in writting of the info in ehealth
●	implementation into medical education\what?\None	none
●	implementation into medical education\what?\None	0
●	implementation into medical education\what?\None	None
●	implementation into medical education\what?\None	None
●	implementation into medical education\what?\None	None
●	implementation into medical education\what?\None	None
●	implementation into medical education\what?\None	None
●	implementation into medical education\what?\None	none
●	implementation into medical education\what?\None	None
●	implementation into medical education\what?\None	None
●	implementation into medical education\what?\None	none

●	implementation into medical education\what?\None	none, I´d like a course demonstrating to the cellphone zombie millenial generation how vulnerable they are, publicizing their whole life and health. It will be used for population control and oppression
●	implementation into medical education\what?\None	None
●	implementation into medical education\what?\None	None
●	implementation into medical education\what?\None	None
●	implementation into medical education\what?\None	none at the moment
●	implementation into medical education\what?\None	None
●	implementation into medical education\what?\None	None
●	implementation into medical education\what?\eHealth technologies\VR simulations\3D anatomy courses	3D anatomy courses
●	implementation into medical education\what?\eHealth technologies\VR simulations\3D anatomy courses	radiology, portable sonography, 3d animation or virtual reality animation of human body (anatomy)
●	implementation into medical education\what?\eHealth technologies\VR simulations	Everyting. from CDSS to holograms, to VR simulations
●	implementation into medical education\what?\eHealth technologies\VR simulations	Usage of visuals, artificial intellegience, virtual reality, more databases and revisioned and appopriate databases for current lessions
●	implementation into medical education\what?\eHealth technologies\VR simulations	Simulation-based learning
●	implementation into medical education\what?\eHealth technologies\TeleHealth\patient monitoring	Big data, Patient monitoring
●	implementation into medical education\what?\eHealth technologies\TeleHealth\patient monitoring	A course where you explore free open source apps or programs that monitor patients,
●	implementation into medical education\what?\eHealth technologies\TeleHealth\patient monitoring	Patient monitoring
●	implementation into medical education\what?\eHealth technologies\TeleHealth\patient monitoring	Data management, monitoring, mobile applications
●	implementation into medical education\what?\eHealth technologies\TeleHealth\patient monitoring	eHealth courses regarding vital signs managment
●	implementation into medical education\what?\eHealth technologies\TeleHealth\patient monitoring	Monitoring apps etc. as a Therapie or a help in diagnose
●	implementation into medical education\what?\eHealth technologies\TeleHealth\patient monitoring	Monitoring patients for example
●	implementation into medical education\what?\eHealth technologies\TeleHealth	Tele health

●	implementation into medical education\what?\eHealth technologies\TeleHealth	telemedical courses
●	implementation into medical education\what?\eHealth technologies\TeleHealth	especially teleHealth courses regarding smartwatches
●	implementation into medical education\what?\eHealth technologies\TeleHealth	limits and use of apps and how to monitor our patients when they're at home
●	implementation into medical education\what?\eHealth technologies\TeleHealth	Long distance monitoring softwares and related coding languages
●	implementation into medical education\what?\eHealth technologies\TeleHealth	Telehealth
●	implementation into medical education\what?\eHealth technologies\TeleHealth	Telemedicine , emesicine , esurgery
●	implementation into medical education\what?\eHealth technologies\TeleHealth	About data bases, telemedicine
●	implementation into medical education\what?\eHealth technologies\TeleHealth	telemedicine,
●	implementation into medical education\what?\eHealth technologies\TeleHealth	computer science, telemedicine
●	implementation into medical education\what?\eHealth technologies\TeleHealth	CDSS, telehealth
●	implementation into medical education\what?\eHealth technologies\TeleHealth	Telemedicine and Telehealth
●	implementation into medical education\what?\eHealth technologies\TeleHealth	telemedicine, all aspects
●	implementation into medical education\what?\eHealth technologies\TeleHealth	telecommunications and AI
●	implementation into medical education\what?\eHealth technologies\TeleHealth	Course on how different eHealth apps work, where you can "meet" a doctor in n app instead of physically going a healt care facility. Many patients have consulted apps when they're unwell and it would be useful to learn about what kind of help you can expect they have received.
●	implementation into medical education\what?\eHealth technologies\TeleHealth	Telehealth
●	implementation into medical education\what?\eHealth technologies\TeleHealth	I would like to learn specifically and practically about the use of e-descriptions and the systems about remote control machines.
●	implementation into medical education\what?\eHealth technologies\TeleHealth	The use of eHealth and specifically mHealth and TeleHealth in medicine, Big Data usage, Correct usage of eHealth technologies serving the patient's best interests.

●	implementation into medical education\what?\eHealth technologies\TeleHealth	Courses about teleHealth and about clinical decisions supported by computers or applications.
●	implementation into medical education\what?\eHealth technologies\TeleHealth	Information on mobile apps, wearables and telemedicine, artificial intelligence, programming skills, software engineering
●	implementation into medical education\what?\eHealth technologies\TeleHealth	The use of telemedicine, in-patient management software systems
●	implementation into medical education\what?\eHealth technologies\TeleHealth	Telemedicine
●	implementation into medical education\what?\eHealth technologies\TeleHealth	Training upon usage of technology at health care (electronic prescription, sharing informations). Discusssion upon ethical limitations of sharing patients' information. Training for Telemedicine (2 groups, one being the patient and the other the medical team, discussing how to approach and decrease the distance between them).
●	implementation into medical education\what?\eHealth technologies\TeleHealth	Familiarization with electronic prescriptions, telemedicine courses and programming languages.
●	implementation into medical education\what?\eHealth technologies\TeleHealth	Overview, teleHealth, Big Data
●	implementation into medical education\what?\eHealth technologies\TeleHealth	Online checkups
●	implementation into medical education\what?\eHealth technologies\TeleHealth	usage of data (how to), telemedicine
●	implementation into medical education\what?\eHealth technologies\TeleHealth	Telecare, E health policy framework, E health devices principles
●	implementation into medical education\what?\eHealth technologies\TeleHealth	Big data, telemedicine
●	implementation into medical education\what?\eHealth technologies\TeleHealth	Telemedicine or Telehealth
●	implementation into medical education\what?\eHealth technologies\robotic applications	robotic applications
●	implementation into medical education\what?\eHealth technologies\mHealth applications	apps, gadgets
●	implementation into medical education\what?\eHealth technologies\mHealth applications	especially teleHealth courses regarding smartwatches
●	implementation into medical education\what?\eHealth technologies\mHealth applications	limits and use of apps and how to monitor our patients when they're at home
●	implementation into medical education\what?\eHealth technologies\mHealth applications	Apps

●	implementation into medical education\what?\eHealth technologies\mHealth applications	wearable and mobile technologies in health,
●	implementation into medical education\what?\eHealth technologies\mHealth applications	mHealth oppurtunities.
●	implementation into medical education\what?\eHealth technologies\mHealth applications	Data management, monitoring, mobile applications
●	implementation into medical education\what?\eHealth technologies\mHealth applications	Info about mobile apps, l
●	implementation into medical education\what?\eHealth technologies\mHealth applications	Apps to use as a medical student.
●	implementation into medical education\what?\eHealth technologies\mHealth applications	mHealth
●	implementation into medical education\what?\eHealth technologies\mHealth applications	How to use learning platforms, medical apps, taking a medic decision based on diagnosis apps etc
●	implementation into medical education\what?\eHealth technologies\mHealth applications	Course on how different eHealth apps work, where you can "meet" a doctor in n app instead of physically going a healt care facility. Many patients have consulted apps when they're unwell and it would be useful to learn about what kind of help you can expect they have received.
●	implementation into medical education\what?\eHealth technologies\mHealth applications	The use of eHealth and specifically mHealth and TeleHealth in medicine, Big Data usage, Correct usage of eHealth technologies serving the patient's best interests.
●	implementation into medical education\what?\eHealth technologies\mHealth applications	Information on mobile apps, wearables and telemedicine, artificial intelligence, programming skills, software engineering
●	implementation into medical education\what?\eHealth technologies\mHealth applications	Casse or mhealth
●	implementation into medical education\what?\eHealth technologies\mHealth applications	eHealth Skills and how to deal with wearables
●	implementation into medical education\what?\eHealth technologies\information systems\Electronic Health Record / Patient Data Management System	Security and privacy on eHealth, Records on eHealth
●	implementation into medical education\what?\eHealth technologies\information systems\Electronic Health Record / Patient Data Management System	Clinical database training, Patient record retrieval training,
●	implementation into medical education\what?\eHealth technologies\information systems\Electronic Health Record / Patient Data Management System	Artificial intelligence, patient data programms (SAP?)

●	implementation into medical education\what?\eHealth technologies\information systems\Electronic Health Record / Patient Data Management System	learning about documenting health information in a way that allows us to participate in contributing to big data if we choose to
●	implementation into medical education\what?\eHealth technologies\information systems\Electronic Health Record / Patient Data Management System	Use of patient record systems and safety
●	implementation into medical education\what?\eHealth technologies\information systems\Electronic Health Record / Patient Data Management System	The use of telemedicine, in-patient management software systems
●	implementation into medical education\what?\eHealth technologies\information systems\Electronic Health Record / Patient Data Management System	AI, policy related in terms of patient records etc
●	implementation into medical education\what?\eHealth technologies\information systems\Electronic Health Record / Patient Data Management System	Hospital Data base managent
●	implementation into medical education\what?\eHealth technologies\information systems\Electronic Health Record / Patient Data Management System	Electronic Health insurance card, Electronic Health Records, how Health Apps work, CDSS, (telemedicine)
●	implementation into medical education\what?\eHealth technologies\information systems\Electronic Health Record / Patient Data Management System	app usage, medical registry,
●	implementation into medical education\what?\eHealth technologies\information systems	Informational systems, how to work with them and hoe thay can help us
●	implementation into medical education\what?\eHealth technologies\information systems	Using artificial intelligence, information technology in a healthcare setting
●	implementation into medical education\what?\eHealth technologies\information systems	Patient data handling and safety, e-healthcare system (in Hungary we just implemented a national system that has all patient records/e-prescriptions, etc that is available for doctors + to dentists + to pharmacists, but we as medical students receive no training on it whatsoever)
●	implementation into medical education\what?\eHealth technologies\information systems	Familiarization with electronic prescriptions, telemedicine courses and programming languages.
●	implementation into medical education\what?\eHealth technologies\information systems	Searching Articles (Ex: Clinical Trials, Basic Research...), APP used for patients data base and how it's used.
●	implementation into medical education\what?\eHealth technologies\examples\new developments	New technologies on the market
●	implementation into medical education\what?\eHealth technologies\examples\new developments	opportunities

●	implementation into medical education\what?\eHealth technologies\examples\new developments	future perpectives,
●	implementation into medical education\what?\eHealth technologies\examples\new developments	Recent technological advances and implementations of eHealth in every domain of medicine.
●	implementation into medical education\what?\eHealth technologies\examples\new developments	Courses which are showing me at least the current possibilities and the future opportunities of ehealth.
●	implementation into medical education\what?\eHealth technologies\examples\new developments	New technology and dealing with patients that already use eHealth by themselves
●	implementation into medical education\what?\eHealth technologies\examples\new developments	Methods used and new trends
●	implementation into medical education\what?\eHealth technologies\examples\new developments	Cours on what possibilities there are.
●	implementation into medical education\what?\eHealth technologies\examples\new developments	Health apps, the future of medicine
●	implementation into medical education\what?\eHealth technologies\examples\new developments	mHealth oppurtunities. Courses on latest eHealth implementations. More courses on Big Data systems in use at the moment.
●	implementation into medical education\what?\eHealth technologies\examples\new developments	New technologies, how to use them. Patient education
●	implementation into medical education\what?\eHealth technologies\examples\new developments	They should teach us how the given program works, how can be teach the usage of these to our future patients, introducing to the newest technologies
●	implementation into medical education\what?\eHealth technologies\examples\new developments	Teach us to use the newest programs and show us the new technologies.
●	implementation into medical education\what?\eHealth technologies\examples\new developments	Training in and knowledge of the current existing options. A look at what options are coming in the near future.
●	implementation into medical education\what?\eHealth technologies\examples\new developments	To keep us updated with all the stuff and technilogies nowadays.
●	implementation into medical education\what?\eHealth technologies\examples\new developments	updates on current programms/usages
●	implementation into medical education\what?\eHealth technologies\examples\new developments	critical and responsible use of data, data security, broadening the horizon of possible applications and possibilities
●	implementation into medical education\what?\eHealth technologies\examples\new developments	up-to-date awareness about fundamental eHealth apps and/ or devices
●	implementation into medical education\what?\eHealth technologies\examples\new developments	Current and future trends in mHealth and eHealth solutions
●	implementation into medical education\what?\eHealth technologies\examples\new developments	Modern developments in medical Artficial Intelligence, Analysis of Big Data

●	implementation into medical education\what?\eHealth technologies\examples\new developments	Ethical discussions, basic programming knowledge, examples of opportunities and already existing tools ...
●	implementation into medical education\what?\eHealth technologies\examples\new developments	Programming applied to health, highlighting theory and applications behind common examples
●	implementation into medical education\what?\eHealth technologies\examples	More examples of how it could be used and more infos about good sites we could redirect our patients to
●	implementation into medical education\what?\eHealth technologies\examples	Apps for patients, support and knwlesge systems for doctors
●	implementation into medical education\what?\eHealth technologies\examples	Medical devices
●	implementation into medical education\what?\eHealth technologies\examples	Big data, clinical apps
●	implementation into medical education\what?\eHealth technologies\Blockchain	blockchain in healthcare
●	implementation into medical education\what?\eHealth technologies\AI\CDSS	Everyting. from CDSS to holograms, to VR simulations
●	implementation into medical education\what?\eHealth technologies\AI\CDSS	CDSS
●	implementation into medical education\what?\eHealth technologies\AI\CDSS	CDSS, computer based eHealth technologies
●	implementation into medical education\what?\eHealth technologies\AI\CDSS	Organisational skills , workflow. Assisted decisionmaking.
●	implementation into medical education\what?\eHealth technologies\AI\CDSS	Cdss
●	implementation into medical education\what?\eHealth technologies\AI\CDSS	Courses on big data, analytics, and how this relates to research and clinical decision making. (Definitely after pre-med. Maybe in the 4th o 5th year.)
●	implementation into medical education\what?\eHealth technologies\AI\CDSS	CDSS, telehealth
●	implementation into medical education\what?\eHealth technologies\AI\CDSS	general IT knowledge, how to handle CDSS
●	implementation into medical education\what?\eHealth technologies\AI\CDSS	How to use learning platforms, medical apps, taking a medic decision based on diagnosis apps etc
●	implementation into medical education\what?\eHealth technologies\AI\CDSS	CDSS
●	implementation into medical education\what?\eHealth technologies\AI\CDSS	Use of big data, artificial intelligence, decision making tools
●	implementation into medical education\what?\eHealth technologies\AI\CDSS	Courses about teleHealth and about clinical decisions supported by computers or applications.

●	implementation into medical education\what?\eHealth technologies\AI\CDSS	Especially CDDS'. because it promises a huge potential
●	implementation into medical education\what?\eHealth technologies\AI\CDSS	Clinical decision support, machine learning, precision medicine.
●	implementation into medical education\what?\eHealth technologies\AI\CDSS	CDSS for example would be a good start.
●	implementation into medical education\what?\eHealth technologies\AI\CDSS	Electronic Health insurance card, Electronic Health Records, how Health Apps work, CDSS, (telemedicine)
●	implementation into medical education\what?\eHealth technologies\AI\CDSS	How toolsto support clinical diagnosis works
●	implementation into medical education\what?\eHealth technologies\AI\CDSS	About decision making and start-up opportunities
●	implementation into medical education\what?\eHealth technologies\AI\CDSS	CDSS
●	implementation into medical education\what?\eHealth technologies\AI\CDSS	Lern more about CDSS.
●	implementation into medical education\what?\eHealth technologies\AI	on the use of AI in medicine.
●	implementation into medical education\what?\eHealth technologies\AI	Usage of visuals, artificial intellegience, virtual reality, more databases and revisioned and appopriate databases for current lessions
●	implementation into medical education\what?\eHealth technologies\AI	Programming, AI, data science (other than stats)
●	implementation into medical education\what?\eHealth technologies\AI	Using artificial intelligence, information technology in a healthcare setting
●	implementation into medical education\what?\eHealth technologies\AI	Programming courses especially concerning data science, introduction to machine learning with focus on image recognition (applicable for example in radiological contexts)
●	implementation into medical education\what?\eHealth technologies\AI	Artificial intelligence, patient data programms (SAP?)
●	implementation into medical education\what?\eHealth technologies\AI	Artificial intelligence programs, clinical trial data handling
●	implementation into medical education\what?\eHealth technologies\AI	Artificial intelligence courses, Ethical issues, Programming courses
●	implementation into medical education\what?\eHealth technologies\AI	telecommunications and AI
●	implementation into medical education\what?\eHealth technologies\AI	Use of big data, artificial intelligence, decision making tools

●	implementation into medical education\what?\eHealth technologies\AI	AI, computer science, big data.
●	implementation into medical education\what?\eHealth technologies\AI	Information on mobile apps, wearables and telemedicine, artificial intelligence, programming skills, software engineering
●	implementation into medical education\what?\eHealth technologies\AI	Modern developments in medical Artficial Intelligence, Analysis of Big Data
●	implementation into medical education\what?\eHealth technologies\AI	AI, policy related in terms of patient records etc
●	implementation into medical education\what?\eHealth technologies\AI	Ai in healthcare and radiology
●	implementation into medical education\what?\eHealth technologies\AI	Artificial Intelligence and Big Data
●	implementation into medical education\what?\eHealth technologies\AI	Working with AI
●	implementation into medical education\what?\anything eHealth related\no preference	No preference
●	implementation into medical education\what?\anything eHealth related	some of them, no matter what type of them
●	implementation into medical education\what?\anything eHealth related	Anything
●	implementation into medical education\what?\anything eHealth related	Any
●	implementation into medical education\what?\anything eHealth related	Any kind
●	implementation into medical education\what?\anything eHealth related	Any
●	implementation into medical education\what?\anything eHealth related	any
●	implementation into medical education\what?\anything eHealth related	All of them
●	implementation into medical education\what?\anything eHealth related	Could be anything
●	implementation into medical education\what?\anything eHealth related	Everything
●	implementation into medical education\what?\anything eHealth related	All of them
●	implementation into medical education\what?\anything eHealth related	All of them

●	implementation into medical education\what?\anything eHealth related	All there is
●	implementation into medical education\what?\anything eHealth related	All possibles
●	implementation into medical education\what?\anything eHealth related	Everything important
●	implementation into medical education\what?\anything eHealth related	All possible
●	implementation into medical education\what?\anything eHealth related	Anyone
●	implementation into medical education\what?\anything eHealth related	Anything
●	implementation into medical education\not useful yet	not yet sensefull, but in future will be more necessary to give an overview to all possabilities
●	implementation into medical education\not useful yet	I'm, I don't know of a specific course would help
●	implementation into medical education\how?\tailored to future job requirements	I would like to learn what is expected from me in the future.
●	implementation into medical education\how?\tailored to future job requirements	The ones that are going to be helpful for my national health system.
●	implementation into medical education\how?\tailored to future job requirements	All necessary for doctors to perform their duties effectively when we graduate and are employed in actual hospitals
●	implementation into medical education\how?\tailored to future job requirements	an introduction to the different branches and their use in our career
●	implementation into medical education\how?\tailored to future job requirements	Just the basics of what to expect later on in my job.
●	implementation into medical education\how?\tailored to future job requirements	good ehaelth technologies for patients
●	implementation into medical education\how?\Subject on its own	more as a subject
●	implementation into medical education\how?\interprofessional courses (with IT specialists)	Ehealth apps exploring, together with medical specialists and IT guides
●	implementation into medical education\how?\interprofessional courses (with IT specialists)	A course combining Psychology, Technology and Health
●	implementation into medical education\how?\interprofessional courses (with IT specialists)	programming, more technical subjects and interships

●	implementation into medical education\how?\by choice	any related with my preferences.
●	implementation into medical education\how?\based on subjects\eSurgery	Telemedicine , emesicine , esurgery
●	implementation into medical education\how?\based on subjects\eSurgery	Radiologic courses, surgical courses
●	implementation into medical education\how?\based on subjects\eHealth in radiology	Ai in healthcare and radiology
●	implementation into medical education\how?\based on subjects\eHealth in radiology	Radiologic courses, surgical courses
●	implementation into medical education\how?\based on subjects\eHealth in psychiatry	Psychology and Psychiatry
●	implementation into medical education\how?\based on subjects\eHealth in cardiology	cardiology
●	implementation into medical education\how?\based on subjects	Based on different diseases
●	implementation into medical education\how?\based on subjects	Problem Oriented training
●	implementation into medical education\already implemented	We have enough
●	implementation into medical education\already implemented	We have it already.
●	implementation into medical education\already implemented	Already have - ICT, eHealth, statistics...
