# Supplementary material for: Perceptions of Digital Health Education Among European Medical Students: Mixed Methods Survey
Source: J Med Internet Res. 2020 Aug 14;22(8):e19827. doi: 10.2196/19827 (PMC7455864; doi:10.2196/19827)

Suppl. figure 1: Flow chart of the participation in the survey

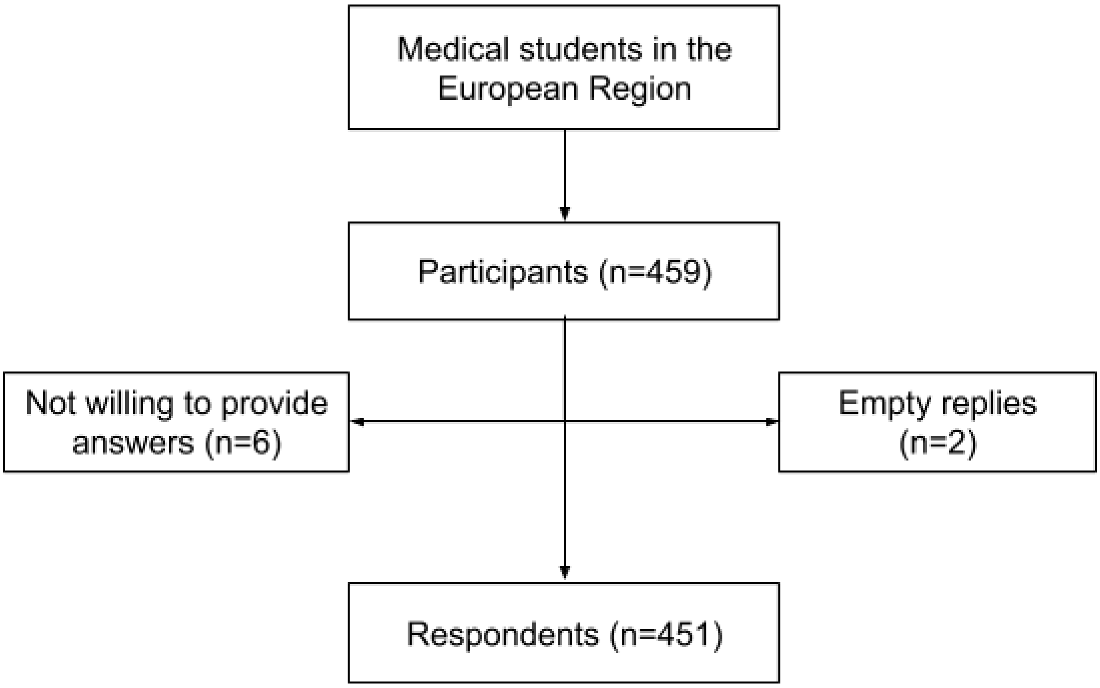

Suppl. figure 2: Overview of year of medical studies of respondents

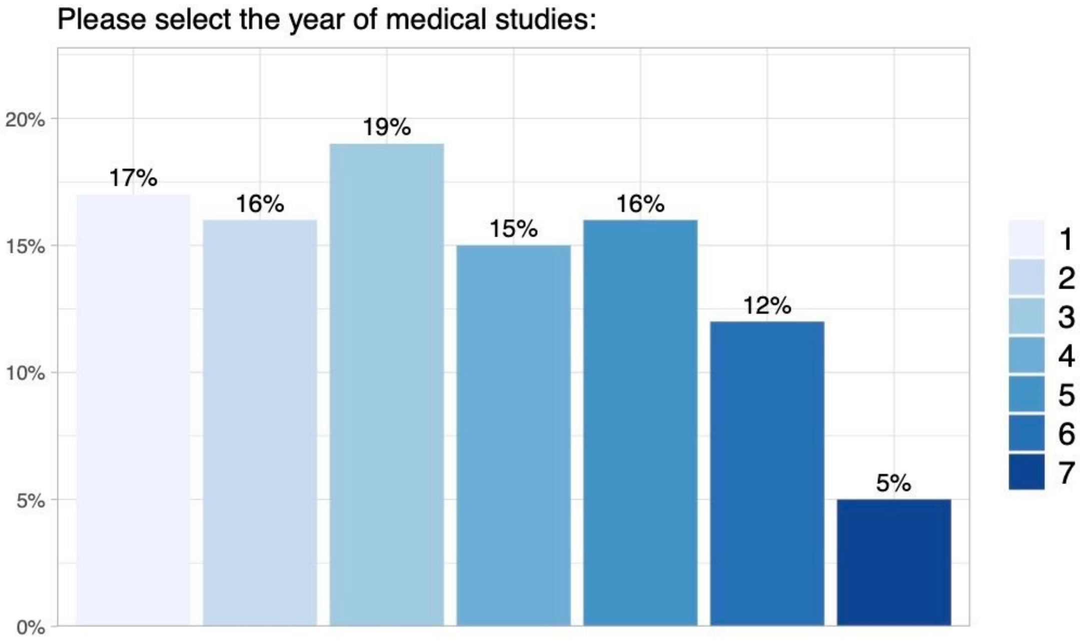

Suppl. figure 3: Geographical distribution of respondents. Countries covered: Albania, Austria, Belgium, Bulgaria, Cyprus, Czech Republic, Denmark, Estonia, Finland, France, Georgia, Germany, Greece, Hungary, Ireland, Italy, Kosovo, Latvia, Lithuania, Luxembourg, Malta, Moldova, Montenegro, Netherlands, Norway, Poland, Portugal, Romania, Russian Federation, Slovakia, Slovenia, Spain, Sweden, Switzerland, the former Yugoslav Republic of Macedonia, Turkey, Ukraine, United Kingdom.

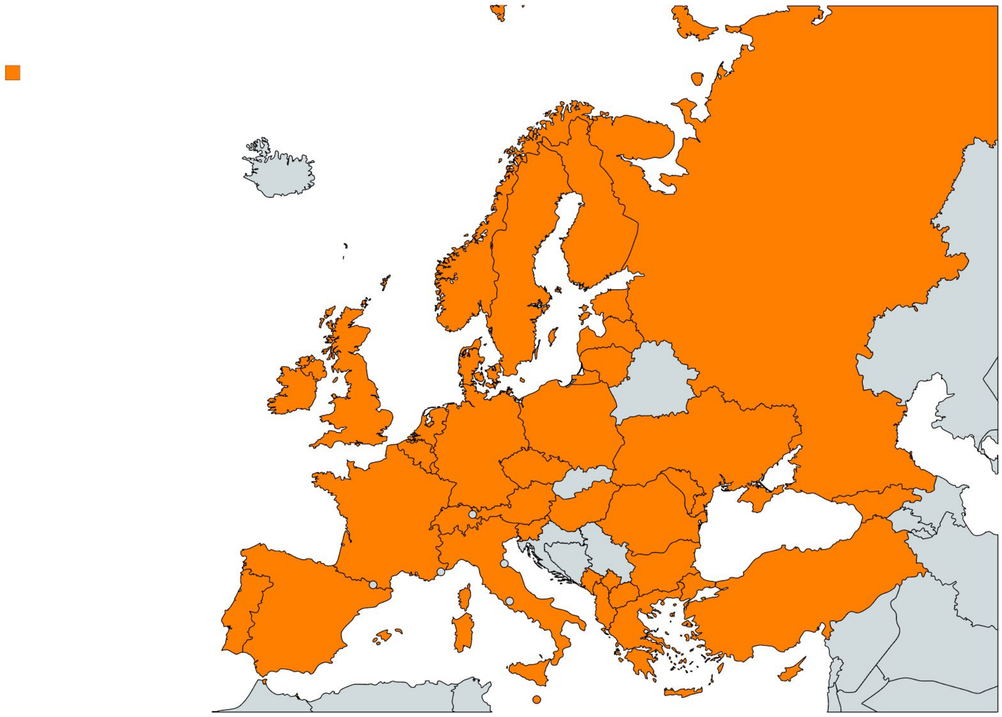

Suppl. figure 4: Attitude of medical students towards mHealth

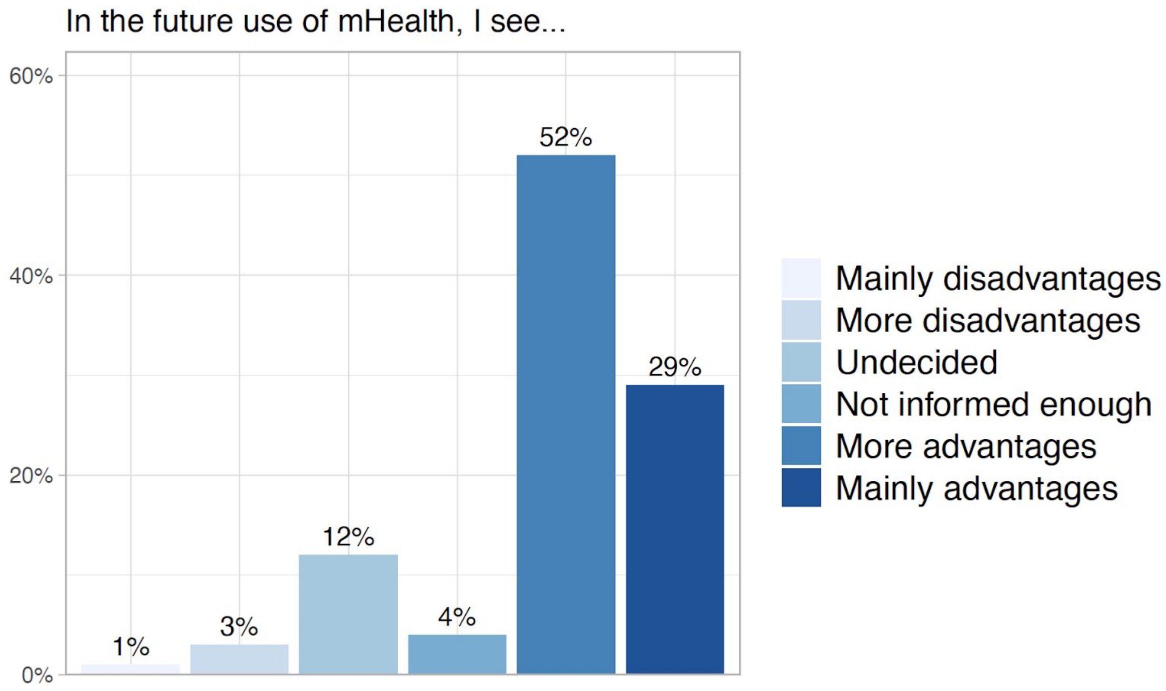

Suppl. figure 5: Attitude of medical students towards teleHealth

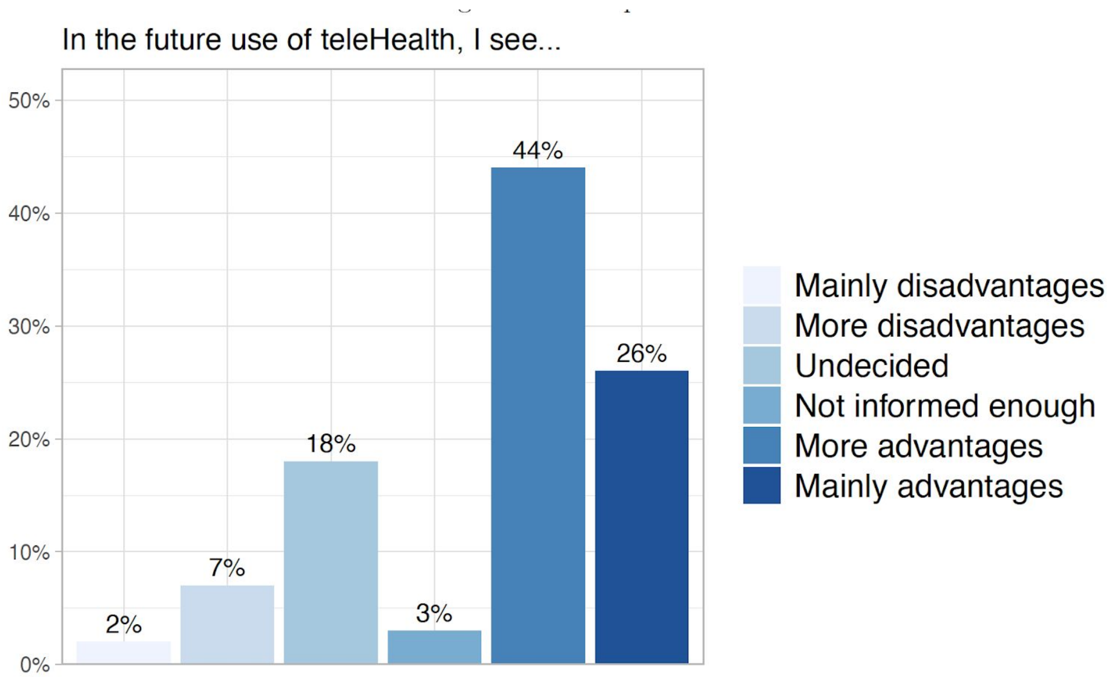

Supp. figure 6: Attitude of medical students towards big data

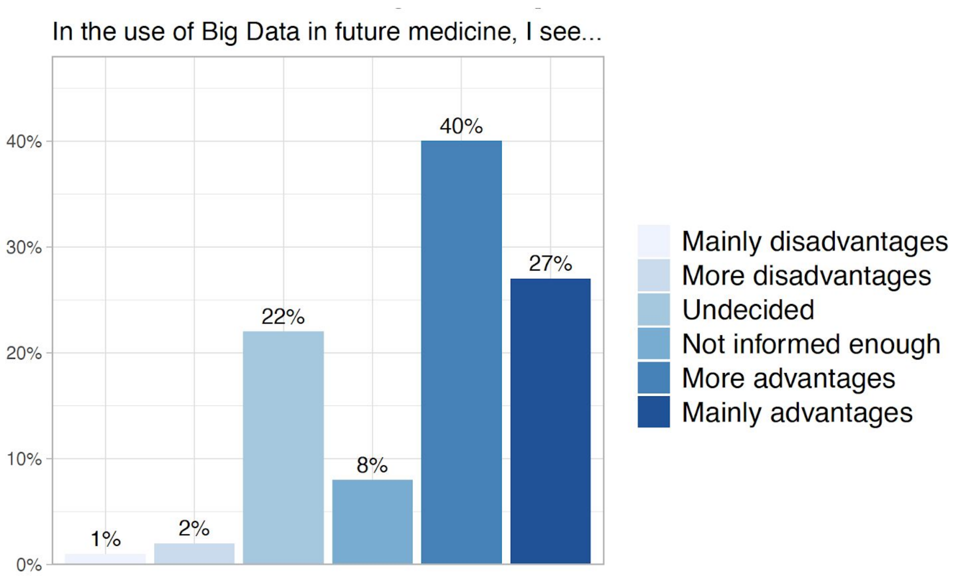

Supplement: Multimedia Appendix 6 [file jmir_v22i8e19827_app6.pdf]
